# Low-Molecular-Weight Chitosan Supplementation Increases the Population of *Prevotella* in the Cecal Contents of Weanling Pigs

**DOI:** 10.3389/fmicb.2017.02182

**Published:** 2017-11-07

**Authors:** Ting Yu, Yu Wang, Shicheng Chen, Min Hu, Zhiling Wang, Guozhong Wu, Xianyong Ma, Zhuang Chen, Chuntian Zheng

**Affiliations:** ^1^Key Laboratory of Animal Nutrition and Feed Science (South China) of Ministry of Agriculture, State Key Laboratory of Livestock and Poultry Breeding, Guangdong Public Laboratory of Animal Breeding and Nutrition, Guangdong Key Laboratory of Animal Breeding and Nutrition, Institute of Animal Science, Guangdong Academy of Agricultural Sciences, Guangzhou, China; ^2^Agro-Biological Gene Research Center, Guangdong Academy of Agricultural Sciences, Guangzhou, China; ^3^Hebei Depond Animal Health Care Science and Technology Co., Ltd, Shijiazhuang, China; ^4^Department of Microbiology and Molecular Genetics, Michigan State University, East Lansing, MI, United States; ^5^Guangdong Key Laboratory of Integrated Agro-Environmental Pollution Control and Management, Guangdong Institute of Eco-Environmental Science & Technology, Guangzhou, China; ^6^Shanghai Institute of Applied Physics, Chinese Academy of Sciences, Shanghai, China

**Keywords:** low-molecular-weight chitosan, weaned piglets, cecal microflora, microbial community, 16S rDNA sequencing

## Abstract

Low-molecular-weight chitosan (LC) promoted growth in weaned piglets as an alternative to feed-grade antibiotics. To investigate the influence of LC supplementation on piglets' gut microbiome and compare the differences in community composition between LC and antibiotics with ZnO addition, we assessed the cecal microbial community by 16S rRNA gene sequencing with three treatments consisting of basal diet (CTR group), basal diet with low-molecular-weight chitosan (LC group), and basal diet with antibiotic and ZnO (AZ group). LC decreased pH more than AZ did in the cecum (both compared to CTR). Beta diversity analysis showed that community structure was distinctly different among the CTR, LC, and AZ treatments, indicating that either LC or AZ treatment modulated the piglet microbiota. *Bacteroidetes, Firmicutes*, and *Proteobacteria* dominated the community [>98% of operational taxonomic units (OTUs)] in piglet cecal contents. Compared to CTR, both LC, and AZ increased the relative abundance of *Bacteroidetes* while they decreased the count of *Firmicutes* and AZ decreased the population of *Proteobacteria*. In CTR the top four abundant genera were *Prevotella* (~10.4%), *Succinivibrio* (~6.2%), *Lactobacillus* (~5.6%), and *Anaerovibri*o (5.4%). Both LC and AZ increased the relative abundance of *Prevotella* but decreased the ratio of *Lactobacillus* when they compared with CTR. Moreover, LC increased the relative abundance of *Succinivibrio* and *Anaerovibri*o while AZ decreased them. The microbial function prediction showed LC enriched more pathways in the metabolism of cofactors and vitamins than CTR or AZ did. LC may potentially function as an alternative to feed-grade antibiotics in weaned piglets due to its beneficial regulation of the intestinal microbiome.

## Introduction

The piglets at an early weaning stage face a dramatic life change in the food source, the immune system as well as the environmental and social status (Pluske et al., 1997; Lallès et al., 2007). These stressful events often cause digestive disorders, nutrient malabsorption and a high incidence of diarrhea in piglets (Madec et al., 1998; Boudry et al., 2004; Fairbrother et al., 2005). Antibiotics and zinc oxide (ZnO) have been widely supplemented into the piglet diets, which improve the growth rates and decrease the diarrhea rates (Barton, 2000; Zhu et al., 2017). However, the continuous addition of antibiotics and ZnO leads to negative consequences including the drug accumulation in livestock products, environmental contamination and bacterial antibiotic resistance (Barton, 2000; Vahjen et al., 2015).

Therefore, alternative diet supplements have been investigated to replace antibiotics and ZnO (Turner et al., 2001). Among the alternative supplements, chitosan (~1,000 kDa of molecular weight) and its derivatives low-molecular-weight chitosan (LC or LMWC, < 150 kDa) or chito-oligosaccharide (COS, < 5 kDa), can be obtained from chitin after the physical, chemical, and enzymatic conversions (Yin et al., 2009). They have been widely applied in chemical, medicinal, food, and agricultural industries such as food and feed additive (Yin et al., 2009; Vinsová and Vavríková, 2011). Due to the properties of non-toxic, biocompatibility and biodegradability as the few alkaline polysaccharides with positive charge (Yin et al., 2009; Vinsová and Vavríková, 2011), they have been reported to possess many beneficial biological properties (e.g., anti-microbial, anti-tumor, anti-oxidant, anti-diabetic, anti-obesity, cholesterol lowering, immunity regulation, and metal ion adsorption in animals; Yin et al., 2009; Vinsová and Vavríková, 2011).

As the lowest molecular weight and the highest water-soluble, COS as a feed additive was more widely studied than chitosan and LC in animals (Jung et al., 2006; Han et al., 2007; Liu et al., 2008, 2010; Yang et al., 2012; Zhou et al., 2012; Kong et al., 2014; Xiao et al., 2014; Xiong et al., 2015). Several studies reported that COS (100~1,000mg/kg) promoted animal growth, increased feed digestibility, reduced the incidence of diarrhea, anti-oxidative, enhanced immunity, and improved intestinal surface barrier function in weaned piglets (Jung et al., 2006; Han et al., 2007; Liu et al., 2008, 2010; Yang et al., 2012; Zhou et al., 2012; Kong et al., 2014; Xiao et al., 2014; Xiong et al., 2015). More importantly, COS protected against pathogenic infections (*Escherichia coli* and *Staphylococcus aureus*) and enhanced commensal bacteria members (*lactobacilli* and *bifidobacteria*) to maintaining the healthy gastrointestinal microflora in animals (Jung et al., 2006; Han et al., 2007; Liu et al., 2008; Yang et al., 2012; Kong et al., 2014). However, a newly reported that low dosage of COS supplementation at 30 mg/kg had no effects on promoting growth performance and even have compromised the intestinal barrier integrity (Xiong et al., 2015). The effects of LC as a feed additive on animals remain largely unknown. However, LC (~12 kDa) had much potent antimicrobial activity than did COS, including against pathogens *E. coli, S. aureus, Pseudomonas aeruginosa, Salmonella enterica* serovar Typhi, and *Bacillus cereus* (Tsai et al., 2004). Moreover, compared to that in COS treatment, diet supplementation of LC increased more lipid metabolism and intestinal disaccharidase activities in obese rats induced by high-fat-diet (Chiu et al., 2017). However, the effects of LC on microbiome profiles in piglets remain unknown. Previous information on gut microbiota affected by the probiotic LC was fragmentary and the investigations were mostly limited in the culture-based method (Tsai et al., 2004; Jung et al., 2006; Han et al., 2007; Liu et al., 2008; Yang et al., 2012; Kong et al., 2014).

Our preliminary data showed that LC (20–30 kDa) at a dosage of 50mg/kg improved the animal growth performance, intestinal tract health and anti-oxidant in weaned piglets (unpublished data). In this study, it was hypothesized that the diets containing LC influenced the piglet gut microbiome and might partly exhibit similar effects as in-feed antibiotics and ZnO. High-throughput sequencing of 16S rRNA gene was performed to investigate the microbial community structure variation of cecal bacteria in the weaned piglets with LC supplement and compared with that in antibiotics and ZnO supplement. The study helps to understand the effects of feed supplement on intestinal bacterial communities and facilitate studies of the alternative strategy for treating diarrhea in piglets. Given the similar gut bacterial communities between human beings and sows, our study here also contributes to understanding the effects of LC supplement on modulating the complexity of animal microbial communities and their functional properties in influencing health and disease.

## Materials and methods

### Animals and sample collection

All experimental procedures were carried out with the approval (IACUC-150701) of the Animal Care and Use Committee in Guangdong Academy of Agricultural Sciences, China. A total of 60 male weaned piglets (Duroc × Landrace × Large White) with an average weight of 6 kg and 21-day old were used in this study. The control (CTR) group was the piglets fed the basal diet (Supplementary Table S1); the antibiotics and ZnO (AZ) treatment group was fed the basal diet supplemented with aureomycin (30 mg/kg), polymyxin E (12 mg/kg) and ZnO (3,000 mg/kg); the LC group was those given the basal diet added with low-molecular-weight (20,000~30,000 Da) chitosan (50 mg/kg), which was provided by Jiaxing Korui Biotech Co. Ltd, Zhejiang, China (http://www.korui-china.com/). The product of LC (KR901, Korui) is fabricated by physical methods and in the form of fine powder, water insoluble but soluble in dilute acidic solution, and recommended dosage 50 g/T for piglet's feed. Five replicates were used for each treatment (4 piglets per replicate, totaling 20 animals). The feeding trials lasted for 28 days. The food and water were daily processed *ad lib*. At day 28, 1 randomly selected piglet in individual replicate for each treatment (total 5 animals/treatment) were slaughtered under anesthesia. The contents of cecum were collected and proceeded. The cecal pH was immediately determined.

### DNA extraction, library construction and 16S rDNA sequencing

Microbial genomic DNA was extracted from 200 mg of the sample using the QIAamp DNA stool minikit (Qiagen, Germany) according to the manufacturer's recommendation. DNA quality was evaluated by the agarose gel electrophoresis. The V4 hypervariable regions (the forward primer was 520F 5-AYTGGGYDTAAAGNG-3 and the reverse primer was 802R 5-TACNVGGGTATCTAATCC-3) of 16S rDNA were PCR amplified from microbial genomic DNA (Caporaso et al., 2011). Briefly, 2 μL of diluted DNA sample (~20 ng/μL) was used for PCR amplification (25 μL mixtures). The PCR conditions were as follows: one pre-denaturation cycle at 98°C for 2 min, 25 cycles of denaturation at 98°C for 15 s, annealing at 50°C for 30 s, and elongation at 72°C for 30 s, and one post-elongation cycle at 72°C for 5 min. The PCR amplicon products were purified using 2% agarose gels and used to construct the sequencing library. The libraries of amplicons were attached to Illumina sequencing adapters using the NEBNext Ultra™ II DNA Library Prep Kit for Illumina (E7645L), purified using AMPure XP beads (Biomek, USA) and quality controlled on an Agilent 2100 Bioanalyzer (Agilent, USA). The pooled libraries were pair-end sequenced on the Illumina MiSeq platform with using 2 × 250 bp MiSeq reagent kit v2 (Illumina, USA).

### DNA sequence analysis

Raw sequences were filtered with the average base quality lower than Q_20_, the quality of the head or tail bases lower than Q_20_, sequence lengths shorter than 150 bp, or reads with N. Then FLASH (v1.2.7) was used to assemble the paired-end sequences (Magoc and Salzberg, 2011). Reads with homopolymer runs exceeding 8 bp, primer mismatches, ambiguous bases and sequence lengths shorter than 150 bp were further removed in QIIME (v1.9.0) (Caporaso et al., 2010). The UCHIME (Chiu et al., 2017) of mothur (v1.31.2) (Edgar et al., 2011) was used to remove the chimera sequences.

Operational taxonomic units (OTUs) were counted for all samples with a cutoff of 97% identity using the UCLUST function (Schloss et al., 2009) in QIIME. Any reads <7 times (0.001%) were removed to minimize the impact of rare OTUs on our data analysis (Edgar and Baterman, 2010). The representative sequences of each clustered OTU were selected according to the maximum length, aligned to Greengenes 16S rRNA gene database (v13.8) (Bokulich et al., 2013) and classified by RDP classifier (v.2.2) (Mc Donald et al., 2012). The alpha-diversity indices (PD, Shannon, observed species and Chao1) were calculated for each sample, and beta-diversity (non-metric multi-dimensional scaling, NMDS) analysis was performed to show the group differences in microbial community structure. Beta-diversity statistical analyses were also tested using permutational multivariate analysis of variance (PERMANOVA) based on Bray–Curtis dissimilarities and 999 permutations in the vegan package (v. 2.3.2) (Wang et al., 2007).

### Microbial function prediction and statistical analysis

The microbial function was predicted using Phylogenetic Investigation of Communities by Reconstruction of Unobserved States (PICRUSt) (v1.1.0) (Oksanen et al., 2015). Based on the pre-calculated Greengenes (v13.5) database (Langille et al., 2013), PICRUSt was performed on the abundance predictions of Kyoto Encyclopedia of Genes and Genomes (KEGG) orthologs and KEGG pathways of bacterial communities. The functional differences among groups were compared using Statistical Analysis of Metagenomic Profiles (STAMP) (Parks et al., 2014). Statistical analysis of two groups was conducted for Two-sided Welch's *t*-test and Benjamini-Hochberg FDR correction. Multiple groups were conducted for ANOVA with Tukey-Kramer test and Benjamini-Hochberg correction. Differences were considered significant at *P* < 0.05. Heatmap diagrams were plotted in R environment (v3.1.2) (http://www.r-project.org).

### Sequence data accession number

Raw paired-end reads were submitted to the Sequence Read Archive of the NCBI (accession number: SRP104359).

## Results

### Effects of LC or AZ on the pH in cecal samples

The cecal pH value in the piglets fed with LC was determined to be 6.19, which was significantly lower than that in the control (6.55) (*P* < 0.05). Similarly, compared to that in the control, the cecal pH (6.33) was significantly lower in the piglets fed with AZ (*P* < 0.05). The pH between LC and AZ treatments was not significantly different (*P* > 0.05) (Supplementary Table S2).

### Characteristics of midgut bacteria community libraries

**Figure 1:**
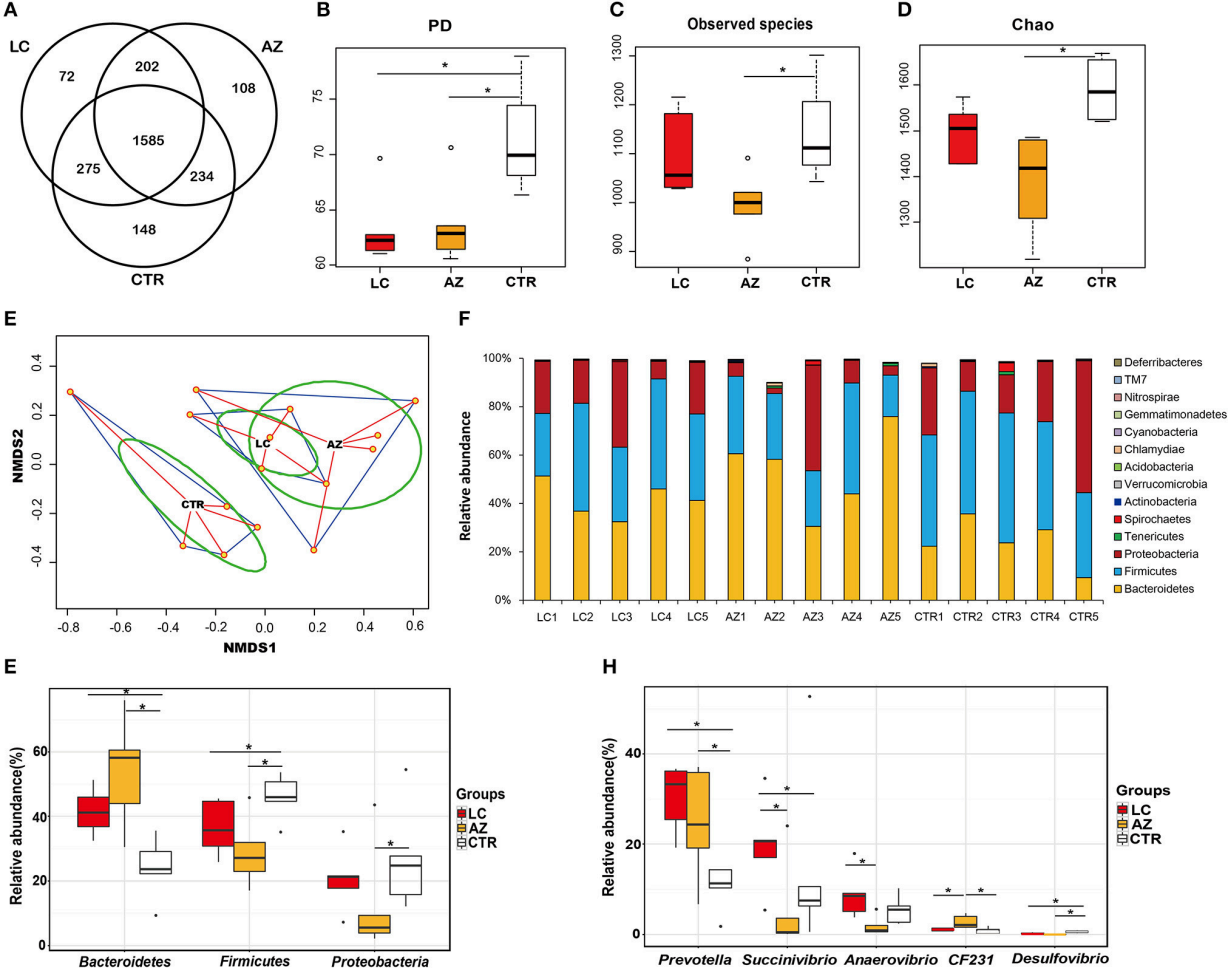
Microbial composition, alpha, and beta-diversity comparison among CTR, AZ and LC groups. **(A)** Comparison of OTUs in the three groups. A Venn diagram was generated to describe the common and unique OTUs among the three groups. **(B)** Bacterial diversity comparison (PD index). LC group (*n* = 5) and AZ group (*n* = 5) both significantly decreased the cecal bacterial diversity (*P* < 0.05). **(C)** Bacterial richness comparison (Observed species index). AZ group significantly decreased the cecal bacterial richness (*P* < 0.05). **(D)** Bacterial richness (Chao index). AZ group significantly decreased the cecal bacterial richness (*P* < 0.05). **(E)** The microbial community structure comparison. The NMDS indicated that it was distinctly different in distribution of microbiota at each group. **(F)** Characterization of communities at the phylum level. Relative abundance of microbial phyla in the ceca of piglets fed the low-molecular-weight chitosan (LC) or antibiotics and ZnO (AZ) diets. **(G)** The significant difference of phyla. Three predominant phyla of *Bacteroidetes, Fimicutes*, and *Proteobacteria* were affected in LC and AZ groups. **(H)** The significant differences of genera. The relative abundance of *Prevotella* were increased by LC or AZ supplementation. Asterisk (^*^) indicates the significant differences between two groups at *P* < 0.05.

At least 47,083 sequence reads were obtained per sample and a total of 706,251 high-quality sequences were used for later analysis (Supplementary Table S3). Based on 97% sequence similarity, these sequences were assigned to 17,890 OTUs. After removing the rare OTUs (lower than 0.001% of total sequences), 2,242, 2,134, and 2,129 OTUs were retained from CTR group, LC group, and AZ group, respectively (Supplementary Table S4). From the Veen analysis of OTU, out of total 2,624 OTUs, 1,585 (~60% of the total OTUs) existed in three groups (Figure 1A). Instead, 148, 72, and 108 unique OTUs were identified in CTR, LC, and AZ groups, respectively.

### Effects of dietary LC and AZ on alpha and beta bacterial diversity

We compared bacterial diversity (PD and Shannon index) and richness (observed species and Chao index) indices for alpha-diversity. PD index in the LC group or AZ group was significantly lower than that in the CTR group (*P* < 0.05), showing that the bacterial diversity in cecum was decreased by AZ or LC (Figure 1B). Shannon index between the two treatments did not show statistical difference while the value in the treatments was lower than the control (Supplementary Table S5). However, there was no significant difference in diversity index (PD and Shannon index) between LC and AZ (*P* > 0.05), indicating both of the treatment strategies decreased bacterial diversity under our testing conditions. About community richness indices (Observed species and Chao), there was no significant difference between LC and CTR, indicating that LC did not affect bacterial richness. However, the number of observed species or Chao index in the AZ group was significantly lower than that in CTR (*P* < 0.05) (Figures 1C,D), indicating that AZ decreased bacterial richness in the cecum. Collectively, our data suggested that piglets fed AZ had a decreased bacterial richness and diversity. Additionally, supplementary LC only decreased the gut bacterial diversity rather than bacterial richness.

For beta diversity analysis, we examined the relationships in cecal microbiome between the control, AZ supplement and LC addition using NMDS. The distribution of microbiota at each group was distinctly clustered separately along principal coordinate (Figure 1E). Moreover, PERMANOVA was used to test the Beta-diversity statistical analysis. The results showed that three groups were distinctly different in the distribution of microbiota (*P* < 0.05) (Table 1), indicating that LC and AZ significantly affected the cecal bacterial structure when compared to CTR.

**Table 1:** Pseudo F table of PERMANOVA analysis based on Bray-Curtis dissimilarities.

**Source of variance**	**Degree of freedom**	***F***	***R*^2^**	***P.adjusted***
Groups^a^	2	2.813	0.319	0.002^*^
**Pairwise comparison**	***F***	***R***^2^	***P***	***P.adjusted***
LC vs. CTR^a^	3.157	0.283	0.009	0.027^*^
AZ vs. CTR^a^	2.689	0.252	0.009	0.027^*^
LC vs. AZ^a^	2.597	0.245	0.031	0.093

a *Based on genus level and n = 5 per group*.

* *Significant P-values (< 0.05) are bolded*.

### Effects of dietary LC and AZ on the composition of the cecal microbiota

Twenty-two bacterial phyla were assigned in the LC, AZ, and CTR groups and 14 of them were shared among them (Supplementary Table S6). *Bacteroidetes, Firmicutes*, and *Proteobacteria* accounted for more than 98% of the total sequence reads (Figure 1F). Approximately 1% of sequences in the cecum samples could not be assigned to a certain rank (unclassified) (Supplementary Table S6), suggesting that there were uncharacterized bacterial taxa existing in the cecal microbiome. The proportion of *Bacteroidetes* in LC and AZ was 1.5- and 1.9-fold higher than that in the CTR group (*P* < 0.05), respectively (Figure 1G). Instead, the proportion of *Firmicutes* in the LC and AZ treatments was around 0.8- and 0.6-fold lower than that in the CTR group (*P* < 0.05), respectively (Figure 1G). The proportion of *Proteobacteria* in the AZ treatment was 0.2-fold lower than that in the CTR group (*P* < 0.05), but in the LC treatment, it was in accordance with CTR group (Figure 1G).

Moreover, 41 bacterial classes (Supplementary Table S7), 68 orders (Supplementary Table S8), 96 families (Supplementary Table S9), and 124 genera (Supplementary Table S10) were assigned to all the three treatments. On the genus level, the relative abundance of *Prevotella* in the LC group (30% of the total bacterial community) and AZ group (29%) was significantly higher than that in the CTR group (10%) (*P* < 0.05) (Figure 1H). Compared to CTR and AZ groups, the relative abundance of *Succinivibrio* was significantly increased by LC treatment (*P* < 0.05) (Figure 1H). Similarly, the relative abundance of *Anaerovibrio* in the LC group was significantly higher than that in AZ group (*P* < 0.05) (Figure 1H). However, the relative abundance of *CF231* in AZ group was significantly higher than that in CTR as well as LC (*P* < 0.05) (Figure 1H). The relative abundance of *Desulfovibrio* in treatments LC and AZ was significantly lower than that in the control group (*P* < 0.05) (Figure 1H).

### Function prediction of the microbial community in the cecum

**Figure 2:**
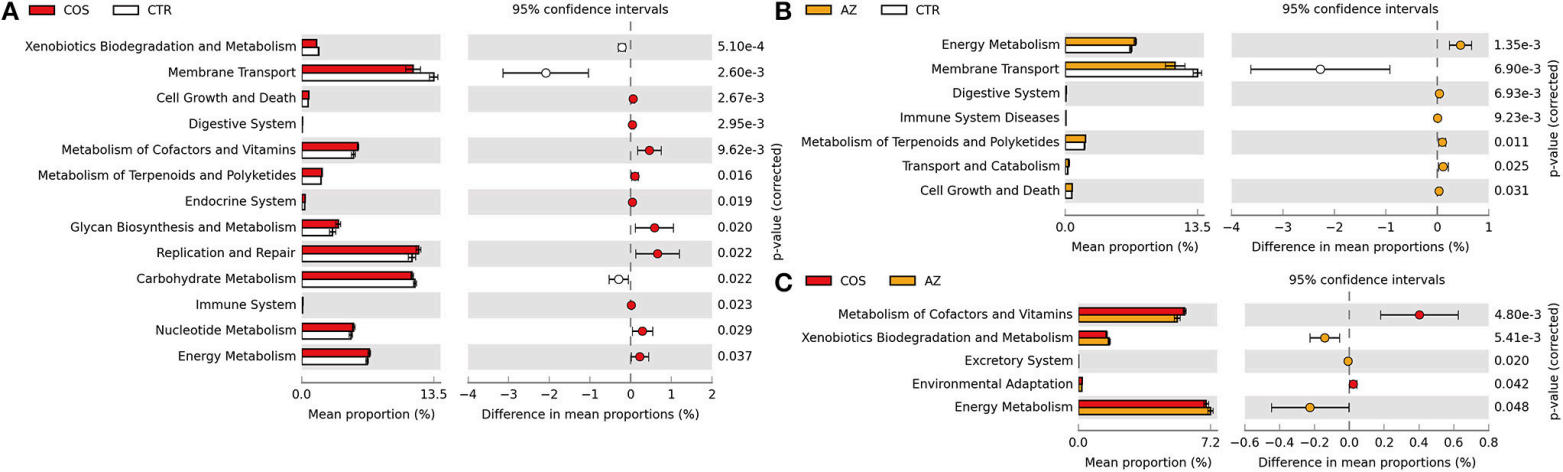
Comparison of microbial function prediction. PICRUSt-predicted relative abundance of KEGG pathway (KEGG level 2) was compared among LC, AZ, and CTR groups. **(A)** the difference of the predicted function between LC and CTR; **(B)** the difference of the predicted function between AZ and CTR; **(C)** the difference of the predicted function between LC and AZ. Statistical analysis was conducted using a Welch's *t*-test between two groups; a significant difference of KEGG pathways (*P.adj* < 0.05) was displayed.

**Figure 3:**
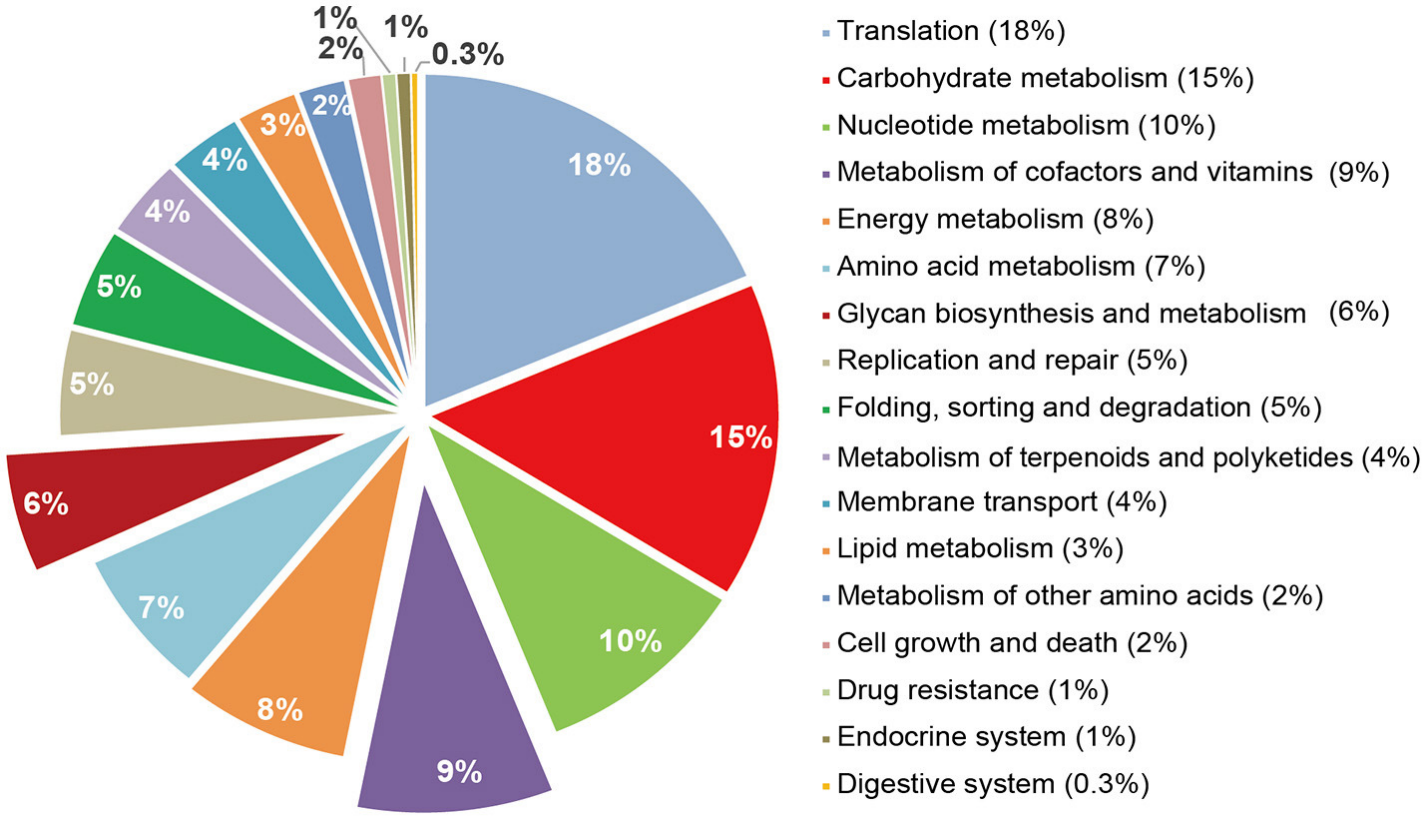
Different KO terms in *Prevotella*. The abundance of enrichments was shown: Translation (18%), Carbohydrate metabolism (15%), Nucleotide metabolism (10%), Metabolism of cofactors and vitamins (9%), Energy metabolism (8%), Amino acid metabolism (7%), Glycan biosynthesis and metabolism (6%).

**Figure 4:**
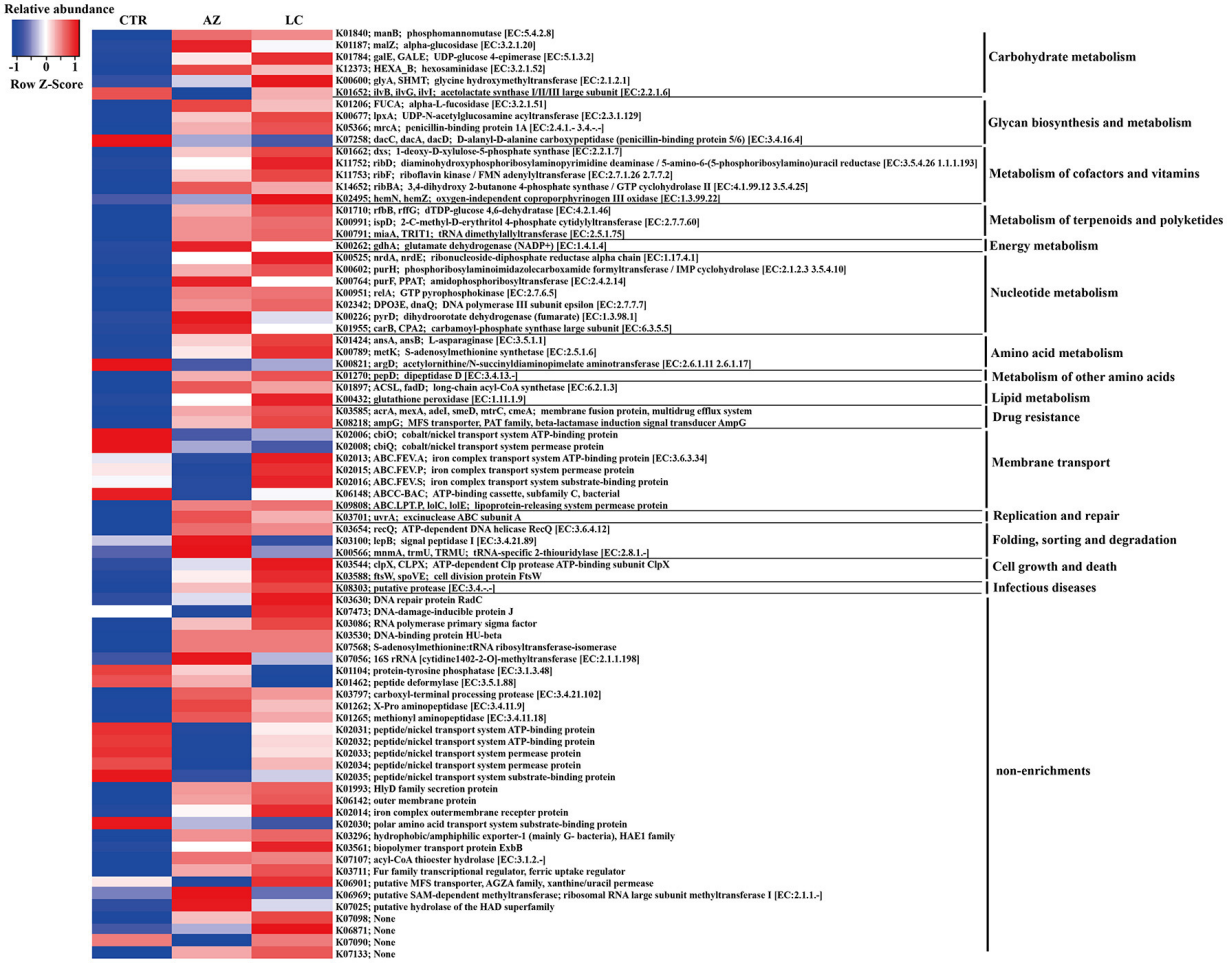
Seventy-nine different KO terms. The relative abundance greater than 0.1% in any group was shown.

Given the distinct microbiome changes within the LC and AZ pigs compared to CTR pigs, we tested whether the different treatments would lead to functional changes in each microbiome. Prediction of metagenome functional contents was conducted by applying PICRUSt to insight our 16S rDNA sequences. Compared to CTR, both LC and AZ had significantly higher function enrichments of energy metabolism, metabolism of terpenoids and polyketides, digestive systems and cell growth and death (*P* < 0.05) (Figures 2A,B). However, both treatments dramatically reduced the function enrichment of environmental information processing such as membrane transport (*P* < 0.05) (Figures 2A,B). In addition, LC group significantly increased the abundance of glycan biosynthesis and metabolism when compared to that in CTR group (*P* < 0.05) (Figure 2A). The relative abundance in the metabolism of cofactors and vitamin in LC treatment was higher than that in CTR or AZ group (*P* < 0.05) (Figures 2A,C). Furthermore, we also analyzed the microbial genes connected to KEGG Orthology (KO) terms. 1,373 of 6,014 KO terms (23%) were found to be significantly different among AZ, LC, and CTR groups based on Tukey-Kramer's ANOVA Test (*P* < 0.05). Remarkably, out of 1,373, 462 KO terms were assigned to bacteria *Prevotella*. It is worthy of highlighting that most of them (462 different KO terms from any two-group comparisons) were enriched on carbohydrate metabolism (15% different KO terms), metabolism of cofactors and vitamins (9%), energy metabolism (8%), glycan biosynthesis and metabolism (6%) (Figure 3), suggesting that the drastically increased *Prevotella* by LC and AZ played an important function among the intestinal microbiota. Further, 79 different KO terms whose relative abundance were >0.1% in any group were screened out, and most of them including iron complex were increased in LC than in CTR group (Figure 4). In the metabolism pathways, there were 62 and 29 different KO terms, whose relative abundance was >0.01% in any group, involved in the metabolism of cofactors and vitamins (Supplementary Figure S1) and glycan biosynthesis and metabolism (Supplementary Figure S2), respectively.

## Discussion

The gut microbiota plays a critical role in animal growth and production; a different influence on microbiome profiles resulting from different treatments is expected to impact host digestion efficiency and intestinal function (Frese et al., 2015). LC, a growth promoter as the alternative supplement in animals, possibly promoted the animal growth performance by modulating the microbiota in the host digestion tract. Our study showed that supplementation of LC (50 mg/kg) to piglets decreased the cecal bacterial diversity without affecting bacterial richness while utilization of antibiotics and ZnO as antimicrobials decreased the cecal bacterial community richness and the bacterial diversity (Supplementary Table S5 and Figures 1B–D). Our early study also observed that in-feed antibiotics or high dietary ZnO supplementation to weaned piglets increased the microbiota diversity and richness of ileal digesta whereas decreased the microbiota diversity of the colonic digesta (Yu et al., 2017). Further, microbial community structure in piglets fed with LC was remarkably different from that in AZ group or CTR (Table 1, Figure 1E). Collectively, our results demonstrated LC differently modulated the intestinal microbiota in piglets using a different mechanism from that in AZ.

In this study, the supplementation of LC (50 mg/kg) to weaned piglets significantly increased the cecal relative abundance of *Bacteroidetes* (Figure 1G). *Bacteroidetes* are well-known excellent degraders for plant polysaccharides and other recalcitrant organic carbon and nitrogen sources (Salyers, 1984). It is also reported that abundant *Bacteroidetes* may prevent diarrhea because members of phylum *Bacteroidetes* are known to interact with the gut immune system, suggesting a link between early microbiota colonization and gut immune maturation after weaning (Jakobsson et al., 2013). Particularly, the abundance of genus *Prevotella* within phylum *Bacteroidetes* was acutely increased from 10.41% in control group to 30.17% in LC group (Supplementary Table S10 and Figure 1H). *Prevotella* are saccharolytic and produce acetate and succinic acids as end fermentation products (Downes et al., 2007). Evidence showed a strong association between *Prevotella* and carbohydrates from fiber-rich diets or from long-term carbohydrates diets (De Filippo et al., 2010; Wu et al., 2011). It was also reported that a higher abundance of *Prevotellaceae* dominated in fecal microbiota of healthy piglets when compared to post-weaning diarrheic piglets (Dou et al., 2017). Thus, diets supplementation of LC, as oligosaccharides that belong to carbohydrates, stimulated the amount increase of *Bacteroidetes* and *Prevotella* to help the breakdown of carbohydrate and maybe improve the intestinal immune and decrease diarrhea. Further investigations are needed to establish the relation between the abundance of *Bacteroidetes* or *Prevotella* and diarrhea. Kong et al. (2014) indicated that dietary supplementation of COS at 500 mg/kg increased the number of *Prevotella* in ileal contents (Kong et al., 2014). Our study here showed that LC supplementation (50 mg/kg) could increase the gastrointestinal *Prevotella* population. Moreover, antibiotics and ZnO supplement also increased the relative abundance of *Prevotella*. The weaned pigs fed with chlortetracycline had an increased *Prevotella* in fecal samples (Li et al., 2017). Collectively, *Prevotella* could be a potential microbial marker for modulation effects of gut microflora by LC or AZ supplementation.

An interesting finding is that the relative abundance of *Succinivibrio* was increased 38-fold in LC group while it was decreased 0.17-fold in AZ group compared to that in CTR (Figure 1H). The relative abundance of *Succinivibrio* in faece was decreased in the weaned pigs fed with chlortetracycline, or zinc bacitracin, or colistin sulfate, or a mixture of them, indicating that *Succinivibrio* was sensitive to antibiotics or zinc (Li et al., 2017). Bacteria *Succinivibrio* are known to degrade starch and produce acetic and succinic acids (Wang et al., 2017). Our results further demonstrated that *Succinivibrio* involving in polysaccharide biodegradation can be increased by some oligosaccharides (i.e., COS). Moreover, the relative abundance of *Enterobacteriaceae* and *Staphylococcus* in the cecum presented a decreased trend in LC group and AZ group (Supplementary Tables S9, S10). However, dietary supplementation with LC (50mg/kg) and AZ group had no effects on increasing *Lactobacillus* and *Bifidobacterium* counts in the cecum (Table S10). The decreased intestinal pH may contribute to inhibit intestinal pathogen propagation and alleviate post-weaning diarrhea in young animals (Mourao et al., 2006). The cecal pH in LC group was the lowest compared to AZ and CTR (Supplementary Table S2). It is reasonable to assume the decreased pH is attributed to the increased *Prevotella* and *Succinivibrio* in LC because both produce acetic and succinic acids as the end fermentation products (Downes et al., 2007; Wang et al., 2017).

Prediction of metagenome functional contents showed that several metabolic pathways were enriched in piglets with LC supplementation including energy metabolism, metabolism of terpenoids and polyketides, digestive systems, cell growth and death, glycan biosynthesis and metabolism as well as metabolism of cofactors and vitamin (Figure 2). It is worthy of highlighting that “digestive systems,” particularly those involved in biodegradation of plant materials (Figure 4), are very important for early-weaned piglets because of the sudden switching from protein-rich milk to plant fiber-rich diets. Abbreviation of such pathways in animal digestion tracts will cause digestive disorders, nutrient malabsorption and a high incidence of diarrhea. Terpenoids and polyketides serve numerous biochemical functions such as quinones in electron transport chains, membrane components, hormones, anti-microbial, and anti-parasites.

Moreover, LC increased the abundances of riboflavin (V_B2_) kinase and iron complex related proteins (Figure 4). LC effectively chelates some vitamins and heavy metals (such as Fe^3+^) (Varma et al., 2004; Sun et al., 2010), which may contribute to solubilize these cofactors and transport them to hosts.

In conclusion, low-molecular-weight chitosan modulated the gut microbial diversity and altered the microbial community in the cecum of weaned piglets, and showed comparable effects to in-feed antibiotics and ZnO additive, especially increased the population of *Prevotella* microbiota in the cecal digesta. The understanding on effects of low-molecular-weight chitosan on intestinal bacterial communities may provide insights into future application of the alternative strategy for treating diarrhea in piglets.

## Author contributions

CZ and ZC conceived and designed the experiments; YW performed the experiments; TY and SC analyzed the data; MH, ZW and XM provided the experimental materials; GW funded the sequencing cost. TY and SC wrote the manuscript. All authors reviewed the manuscript.

### Conflict of interest statement

The authors declare that the research was conducted in the absence of any commercial or financial relationships that could be construed as a potential conflict of interest.
