# Supplementary material for: Low-Molecular-Weight Chitosan Supplementation Increases the Population of Prevotella in the Cecal Contents of Weanling Pigs
Source: Front Microbiol. 2017 Nov 7;8:2182. doi: 10.3389/fmicb.2017.02182 (PMC5682002; doi:10.3389/fmicb.2017.02182)
Supplement: Supplementary file 1 [file Presentation1.PDF]

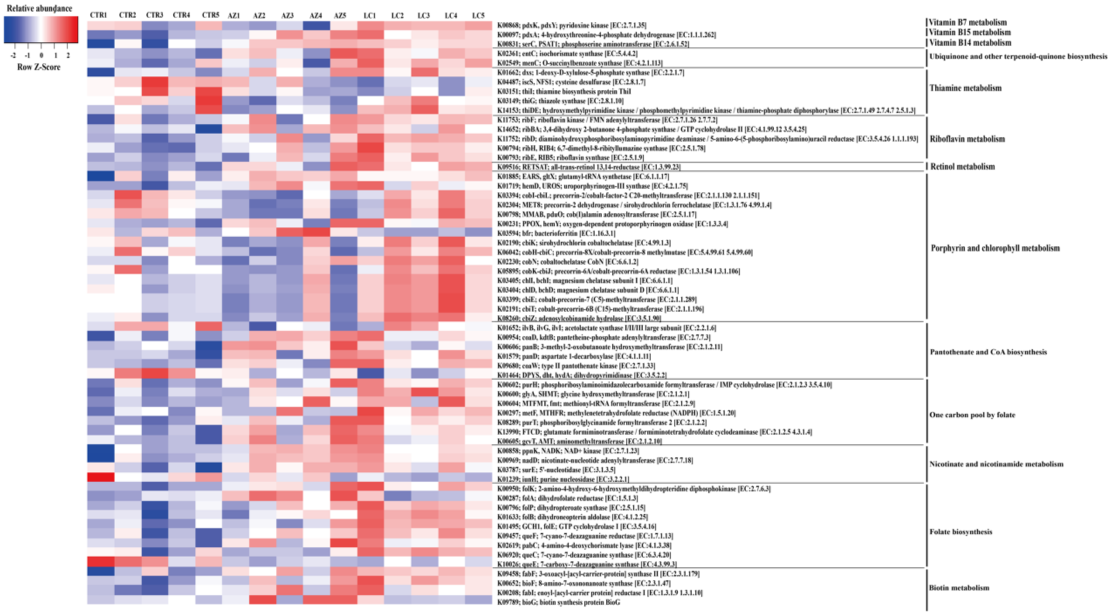
Relative abundance
-2 -1 0 1 2
Row Z-Score
CTR1 CTR2 CTR3 CTR4 CTR5 AZ1 AZ2 AZ3 AZ4 AZ5 LC1 LC2 LC3 LC4 LC5
K00868; pdxK, pdxY; pyridoxine kinase [EC:2.7.1.35]
K00097; pdxA; 4-hydroxythreonine-4-phosphate dehydrogenase [EC:1.1.1.262]
K00831; serC, PSAT1; phosphoserine aminotransferase [EC:2.6.1.52]
K02361; entC; isochorismate synthase [EC:5.4.4.2]
K02549; menC; O-succinylbenzoate synthase [EC:4.2.1.113]
K01662; dxs; 1-deoxy-D-xylulose-5-phosphate synthase [EC:2.2.1.7]
K04487; iscS, NFS1; cysteine desulfurase [EC:2.8.1.7]
K03151; thiI; thiamine biosynthesis protein ThiI
K03149; thiG; thiazole synthase [EC:2.8.1.10]
K14153; thiDE; hydroxymethylpyrimidine kinase / phosphomethylpyrimidine kinase / thiamine-phosphate diphosphorylase [EC:2.7.1.49 2.7.4.7 2.5.1.3]
K11753; ribF; riboflavin kinase / FMN adenylyltransferase [EC:2.7.1.26 2.7.7.2]
K14652; ribBA; 3,4-dihydroxy 2-butanone 4-phosphate synthase / GTP cyclohydrolase II [EC:4.1.99.12 3.5.4.25]
K11752; ribD; diaminohydroxyphosphoribosylaminopyrimidine deaminase / 5-amino-6-(5-phosphoribosylamino)uracil reductase [EC:3.5.4.26 1.1.1.193]
K00794; ribH, RIB4; 6,7-dimethyl-8-ribityllumazine synthase [EC:2.5.1.78]
K00793; ribE, RIB5; riboflavin synthase [EC:2.5.1.9]
K09516; RETSAT; all-trans-retinol 13,14-reductase [EC:1.3.99.23]
K01885; EARS, gltX; glutamyl-tRNA synthetase [EC:6.1.1.17]
K01719; hemD, UROS; uroporphyrinogen-III synthase [EC:4.2.1.75]
K03394; cobI-cbiL; precorrin-2/cobalt-factor-2 C20-methyltransferase [EC:2.1.1.130 2.1.1.151]
K02304; MET8; precorrin-2 dehydrogenase / sirohydrochlorin ferrochelatase [EC:1.3.1.76 4.99.1.4]
K00798; MMAB, pduO; cob(I)alamin adenosyltransferase [EC:2.5.1.17]
K00231; PPOX, hemY; oxygen-dependent protoporphyrinogen oxidase [EC:1.3.3.4]
K03594; bfr; bacterioferritin [EC:1.16.3.1]
K02190; cbiK; sirohydrochlorin cobaltochelatase [EC:4.99.1.3]
K06042; cobH-cbiC; precorrin-8X/cobalt-precorrin-8 methylmutase [EC:5.4.99.61 5.4.99.60]
K02230; cobN; cobaltochelatase CobN [EC:6.6.1.2]
K05895; cobK-cbiJ; precorrin-6A/cobalt-precorrin-6A reductase [EC:1.3.1.54 1.3.1.106]
K03405; chlI, bchI; magnesium chelatase subunit I [EC:6.6.1.1]
K03404; chlD, bchD; magnesium chelatase subunit D [EC:6.6.1.1]
K03399; cbiE; cobalt-precorrin-7 (C5)-methyltransferase [EC:2.1.1.289]
K02191; cbiT; cobalt-precorrin-6B (C15)-methyltransferase [EC:2.1.1.196]
K08260; cbiZ; adenosylcobinamide hydrolase [EC:3.5.1.90]
K01652; ilvB, ilvG, ilvI; acetolactate synthase I/II/III large subunit [EC:2.2.1.6]
K00954; coaD, kdtB; pantetheine-phosphate adenylyltransferase [EC:2.7.7.3]
K00606; panB; 3-methyl-2-oxobutanoate hydroxymethyltransferase [EC:2.1.2.11]
K01579; panD; aspartate 1-decarboxylase [EC:4.1.1.11]
K09680; coaW; type II pantothenate kinase [EC:2.7.1.33]
K01464; DPYS, dht, hydA; dihydropyrimidinase [EC:3.5.2.2]
K00602; purH; phosphoribosylaminoimidazolecarboxamide formyltransferase / IMP cyclohydrolase [EC:2.1.2.3 3.5.4.10]
K00600; glyA, SHMT; glycine hydroxymethyltransferase [EC:2.1.2.1]
K00604; MTFMT, fmt; methionyl-tRNA formyltransferase [EC:2.1.2.9]
K00297; metF, MTHFR; methylenetetrahydrofolate reductase (NADPH) [EC:1.5.1.20]
K08289; purT; phosphoribosylglycinamide formyltransferase 2 [EC:2.1.2.2]
K13990; FTCD; glutamate formiminotransferase / formiminotetrahydrofolate cyclodeaminase [EC:2.1.2.5 4.3.1.4]
K00605; gcvT, AMT; aminomethyltransferase [EC:2.1.2.10]
K00858; ppnK, NADK; NAD+ kinase [EC:2.7.1.23]
K00969; nadD; nicotinate-nucleotide adenylyltransferase [EC:2.7.7.18]
K03787; surE; 5'-nucleotidase [EC:3.1.3.5]
K01239; iunH; purine nucleosidase [EC:3.2.2.1]
K00950; folK; 2-amino-4-hydroxy-6-hydroxymethyldihydropteridine diphosphokinase [EC:2.7.6.3]
K00287; folA; dihydrofolate reductase [EC:1.5.1.3]
K00796; folP; dihydropteroate synthase [EC:2.5.1.15]
K01633; folB; dihydroneopterin aldolase [EC:4.1.2.25]
K01495; GCH1, folE; GTP cyclohydrolase I [EC:3.5.4.16]
K09457; queF; 7-cyano-7-deazaguanine reductase [EC:1.7.1.13]
K02619; pabC; 4-amino-4-deoxychorismate lyase [EC:4.1.3.38]
K06920; queC; 7-cyano-7-deazaguanine synthase [EC:6.3.4.20]
K10026; queE; 7-carboxy-7-deazaguanine synthase [EC:4.3.99.3]
K09458; fabF; 3-oxoacyl-[acyl-carrier-protein] synthase II [EC:2.3.1.179]
K00652; bioF; 8-amino-7-oxononanoate synthase [EC:2.3.1.47]
K00208; fabI; enoyl-[acyl-carrier protein] reductase I [EC:1.3.1.9 1.3.1.10]
K09789; bioG; biotin synthesis protein BioG
Vitamin B7 metabolism
Vitamin B15 metabolism
Vitamin B14 metabolism
Ubiquinone and other terpenoid-quinone biosynthesis
Thiamine metabolism
Riboflavin metabolism
Retinol metabolism
Porphyrin and chlorophyll metabolism
Pantothenate and CoA biosynthesis
One carbon pool by folate
Nicotinate and nicotinamide metabolism
Folate biosynthesis
Biotin metabolism

Figure S1

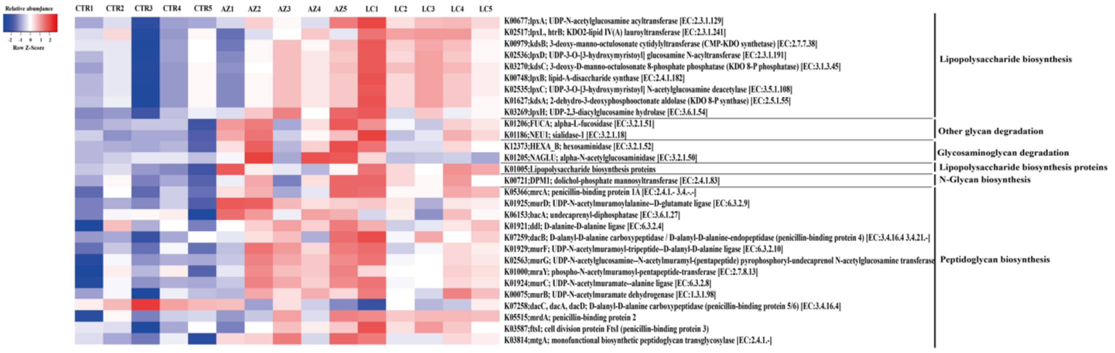

Figure S2

Table S1 | The formulation of diets and nutrient content (%).

Ingredients	%	Nutrition level	%
Corn（8.24%CP）	60.43	CP	19.55
Extruded soybean meal（45.30%CP）	20.00	DE，MJ/kg	14.10
Fish meal（69.14%CP）	5.00	Lys	1.17
Soybean protein concentrate（65%CP）	2.00	Met	0.50
Whey powder（6.13%CP）	7.50	M+C	0.74
Soybean oil	1.50	Thr	0.77
L-Lysine.HCl（78% L-lysine）	0.25	Ca	0.86
DL-Met	0.10	AP	0.68
CaHPO4	1.05		
Limestone	0.75		
50% Choline Chloride	0.12		

NaCl	0.30
Premix	1.00
Total	100.00

Note. 1. Premix contains per kg: vitamin A 11000IU; vitamin D_3 1100IU, vitamin E 80IU,vitamin $K_3$2.5mg, vitamin $B_1$7.5mg, vitamin $B_2$20mg, vitamin $B_6$10mg, vitamin B_{12}220ug, nicotinamide 150mg, Ca-pantithenate 1.5mg, folacin 1.5mg, biotin 3mg, Fe 150mg, Cu 10mg, Mn 10mg, Zn 150mg, I 0.2mg, Se 0.3mg, Co 0.15mg.

2. The crude protein value was actual measured value and the others were calculated valued.

Table S2 | Measurement of pH values in the cecal contents.

	**COS**[a]	**AZ**[a]	**CTR**[a]	**P (COS vs CTR)**[b]	**P (AZ vs CTR)** [b]	**P (COS vs AZ)** [b]
pH	6.19±0.23	6.33±0.13	6.55±0.15	**0.04**	**0.03**	0.36

[a] Five replicates were used for one treatment.

[b] Statistical analysis was conducted using T-test. Significant *P*-values (< 0.05) are bolded.

Table S3 | The reads of each sample in cecal contents.

Samples	Raw reads	Clean reads	Ratio
AZ-1	66,726	57,527	86.21%
AZ-2	58,823	45,016	76.53%
AZ-3	60,355	50,733	84.06%
AZ-4	56,185	48,197	85.78%
AZ-5	77,844	54,162	69.58%
CTR-1	54,223	40,141	74.03%
CTR-2	71,450	53,727	75.20%
CTR-3	69,123	49,794	72.04%
CTR-4	47,842	39,265	82.07%
CTR-5	45,963	37,296	81.14%
LC-1	65,359	52,024	79.60%
LC-2	62,145	47,087	75.77%
LC-3	53,773	44,129	82.07%
LC-4	62,443	47,279	75.72%
LC-5	51,403	39,874	77.57%
Total	903,657	706,251	78.15%

Summary	
Num samples	15
Total Reads	706,251
Min reads/sample	37,296
Max reads/sample	57,527
Mean reads/sample	47,083

Table S4 | The OTU numbers of each group in cecal contents (n=5 per group).

Groups	Cecal contents
CTR group	2,242
AZ group	2,134
LC group	2,129
Total OTU in all groups	2,627
Common OTU in all groups	1,585(10%)

The OTUs were identified based on 97% sequence similarity and at least seven reads of total samples supported per OTU.

Table S5 | Estimation of richness and diversity indices in cecal microbiota.

	Diversity indices		Richness indices	
**Groups**	**PD**	**Shannon**	**Observed species**	**Chao**
LC [a]	63.4±3.56	5.74±0.31	1102.72±89.25	1493.83±65.03
AZ [a]	63.81±3.97	5.71±0.43	994.9±74.53	1382.2±115.3
CTR [a]	71.29±5.35	6.35±0.76	1141.5±111.01	1589.48±76.16
P (LC vs CTR) [b]	**0.03**	0.14	0.57	0.08
P (AZ vs CTR) [b]	**0.04**	0.15	**0.04**	**0.01**
P (LC vs AZ) [b]	0.86	0.87	0.07	0.09

[a] Five replicates were used for one treatment.

[b] Statistical analysis was conducted using T-test. Significant *P*-values (< 0.05) are bolded.

Table S6 | The phylum composition among AZ, LC and CTR (n=5 per group).

Phylum	Group	LC	AZ	CTR	P (LC vs CTR)	P (AZ vs CTR)	P (LC vs AZ)
Bacteroidetes	AZ-CTR-LC	41.54±7.42%	53.84±17.28%	24.01±9.75%	0.01	0.01	0.18
Firmicutes	AZ-CTR-LC	36.53±8.55%	29.03±10.87%	46.06±7.10%	0.04	0.02	0.26
Proteobacteria	AZ-CTR-LC	20.61±10.06%	12.93±17.34%	27.00±16.64%	0.48	0.03	0.42
Tenericutes	AZ-CTR-LC	0.16±0.11%	0.49±0.43%	0.39±0.54%	0.38	0.74	0.13
Spirochaetes	AZ-CTR-LC	0.22±0.22%	0.45±0.67%	0.75±1.57%	0.48	0.70	0.49
Actinobacteria	AZ-CTR-LC	0.01±0.01%	0.08±0.10%	0.11±0.11%	0.10	0.73	0.18
Verrucomicrobia	AZ-CTR-LC	0.02±0.02%	0.08±0.06%	0.10±0.09%	0.11	0.74	0.08
Acidobacteria	AZ-CTR-LC	0.01±0.00%	0.04±0.03%	0.02±0.01%	0.04	0.20	0.06
Chlamydiae	AZ-CTR-LC	0.08±0.06%	0.30±0.52%	0.30±0.54%	0.38	0.99	0.37
Cyanobacteria	AZ-CTR-LC	0.15±0.12%	0.04±0.06%	0.18±0.17%	0.74	0.11	0.10
Gemmatimonadetes	AZ-CTR-LC	0.00±0.00%	0.03±0.02%	0.01±0.01%	0.01	0.22	0.05
Nitrospirae	AZ-CTR-LC	0.00±0.00%	0.00±0.00%	0.01±0.01%	0.13	0.44	0.03
TM7	AZ-CTR-LC	0.01±0.01%	0.00±0.00%	0.01±0.01%	0.77	0.26	0.24
Deferribacteres	AZ-CTR-LC	0.00±0.00%	0.00±0.00%	0.00±0.01%	0.44	0.53	0.70
Fusobacteria	AZ-CTR	0.00±0.00%	0.00±0.00%	0.10±0.20%	0.29	0.29	0.35
Chloroflexi	AZ-CTR	0.00±0.00%	0.02±0.03%	0.00±0.00%	0.35	0.24	0.22
Fibrobacteres	CTR-LC	0.01±0.01%	0.00±0.00%	0.00±0.01%	0.50	0.28	0.09
Synergistetes	CTR-LC	0.01±0.01%	0.00±0.00%	0.02±0.02%	0.29	0.09	0.14
WPS-2	CTR-LC	0.00±0.00%	0.00±0.00%	0.05±0.12%	0.35	0.34	0.35
WS3	AZ	0.00±0.00%	0.01±0.01%	0.00±0.00%	1	0.27	0.27
[Thermi]	AZ	0.00±0.00%	0.00±0.00%	0.00±0.00%	1	0.18	0.18
Planctomycetes	CTR	0.00±0.00%	0.00±0.00%	0.01±0.01%	0.10	0.10	1
Unassigned	AZ-CTR-LC	0.64±0.25%	2.67±4.16%	0.87±0.69%	0.52	0.37	0.31

Table S7 | The class composition among AZ, LC and CTR (n=5 per group).

Class	Group	LC	AZ	CTR	P (LC vs CTR)	P (AZ vs CTR)	P (LC vs AZ)
Bacteroidia	AZ-CTR-LC	41.52±7.42%	53.65±17.31%	27.61±6.06%	0.02	0.03	0.19
Clostridia	AZ-CTR-LC	33.68±9.63%	23.00±7.73%	35.96±10.12%	0.72	0.05	0.09
Gammaproteobacteria	AZ-CTR-LC	20.19±10.19%	6.28±10.33%	23.01±18.09%	0.77	0.11	0.06
Bacilli	AZ-CTR-LC	1.50±2.35%	1.07±0.97%	5.80±6.00%	0.17	0.12	0.72
Erysipelotrichi	AZ-CTR-LC	1.36±0.76%	4.96±4.51%	4.29±2.78%	0.05	0.79	0.12
Spirochaetes	AZ-CTR-LC	0.22±0.22%	0.45±0.67%	0.75±1.57%	0.48	0.7	0.49
Betaproteobacteria	AZ-CTR-LC	0.19±0.11%	6.49±7.54%	2.73±3.57%	0.15	0.34	0.1
Deltaproteobacteria	AZ-CTR-LC	0.19±0.23%	0.02±0.03%	0.89±0.69%	0.07	0.02	0.14
Mollicutes	AZ-CTR-LC	0.16±0.11%	0.49±0.43%	0.39±0.54%	0.38	0.74	0.13
4C0d-2	AZ-CTR-LC	0.15±0.12%	0.03±0.06%	0.18±0.17%	0.74	0.1	0.08
Chlamydiia	AZ-CTR-LC	0.08±0.06%	0.30±0.52%	0.30±0.54%	0.38	0.99	0.37
Epsilonproteobacteria	AZ-CTR-LC	0.03±0.01%	0.02±0.04%	0.32±0.37%	0.12	0.11	0.77
Alphaproteobacteria	AZ-CTR-LC	0.02±0.01%	0.12±0.11%	0.06±0.03%	0.03	0.25	0.08
Coriobacteriia	AZ-CTR-LC	0.01±0.01%	0.02±0.01%	0.06±0.06%	0.08	0.13	0.13
Cytophagia	AZ-CTR-LC	0.01±0.00%	0.10±0.11%	0.02±0.02%	0.09	0.17	0.09
Sphingobacteriia	AZ-CTR-LC	0.01±0.01%	0.02±0.02%	0.01±0.01%	0.41	0.29	0.09
TM7-3	AZ-CTR-LC	0.01±0.01%	0.00±0.00%	0.01±0.01%	0.77	0.26	0.24
Verruco-5	AZ-CTR-LC	0.01±0.02%	0.00±0.00%	0.06±0.08%	0.22	0.13	0.26
Verrucomicrobiae	AZ-CTR-LC	0.01±0.00%	0.05±0.04%	0.03±0.02%	0.04	0.36	0.06
Acidobacteria-6	AZ-CTR-LC	0.00±0.00%	0.01±0.01%	0.00±0.00%	0.32	0.2	0.12
Acidobacteriia	AZ-CTR-LC	0.00±0.00%	0.00±0.00%	0.00±0.00%	0.83	0.23	0.27
[Chloracidobacteria]	AZ-CTR-LC	0.00±0.00%	0.02±0.02%	0.01±0.01%	0.02	0.28	0.05
Actinobacteria	AZ-CTR-LC	0.00±0.00%	0.06±0.10%	0.04±0.06%	0.21	0.66	0.19
Flavobacteriia	AZ-CTR-LC	0.00±0.00%	0.02±0.02%	0.01±0.01%	0.17	0.28	0.07
[Saprospirae]	AZ-CTR-LC	0.00±0.00%	0.04±0.03%	0.02±0.01%	0.03	0.16	0.02
Chloroplast	AZ-CTR-LC	0.00±0.00%	0.00±0.01%	0.00±0.00%	0.85	0.41	0.39
Deferribacteres	AZ-CTR-LC	0.00±0.00%	0.00±0.00%	0.00±0.01%	0.44	0.53	0.7
Gemm-1	AZ-CTR-LC	0.00±0.00%	0.00±0.00%	0.00±0.00%	0.23	0.3	0.13
Gemmatimonadetes	AZ-CTR-LC	0.00±0.00%	0.02±0.02%	0.01±0.01%	0.02	0.23	0.05
Nitrospira	AZ-CTR-LC	0.00±0.00%	0.00±0.00%	0.01±0.01%	0.13	0.44	0.03
[Spartobacteria]	AZ-CTR-LC	0.00±0.00%	0.03±0.02%	0.01±0.01%	0.17	0.15	0.03
Ellin6529	AZ-CTR	0.00±0.00%	0.01±0.02%	0.00±0.00%	0.35	0.21	0.17
Fusobacteriia	AZ-CTR	0.00±0.00%	0.00±0.00%	0.10±0.20%	0.29	0.29	0.35
iii1-8	AZ-CTR	0.00±0.00%	0.00±0.00%	0.00±0.00%	0.35	0.19	0.08
Fibrobacteria	CTR-LC	0.01±0.01%	0.00±0.00%	0.00±0.01%	0.5	0.28	0.09
Synergistia	CTR-LC	0.01±0.01%	0.00±0.00%	0.02±0.02%	0.29	0.09	0.14
Chloroflexi	AZ	0.00±0.00%	0.01±0.01%	0.00±0.00%	1	0.31	0.31
Opitutae	AZ	0.00±0.00%	0.00±0.00%	0.00±0.00%	1	0.08	0.08
PRR-12	AZ	0.00±0.00%	0.01±0.01%	0.00±0.00%	1	0.27	0.27
Deinococci	AZ	0.00±0.00%	0.00±0.00%	0.00±0.00%	1	0.18	0.18
Planctomycetia	CTR	0.00±0.00%	0.00±0.00%	0.01±0.01%	0.1	0.1	1
Unassigned	AZ-CTR-LC	0.64±0.25%	2.67±4.16%	0.87±0.69%	0.52	0.37	0.31

Table S8 | The order composition among AZ, LC and CTR (n=5 per group).

Order	Group	LC	AZ	CTR	P (LC vs CTR)	P (AZ vs CTR)	P (LC vs AZ)
Bacteroidales	AZ-CTR-LC	41.52±7.42%	53.65±17.31%	27.61±6.06%	0.02	0.03	0.19
Clostridiales	AZ-CTR-LC	33.68±9.63%	23.00±7.73%	35.96±10.12%	0.72	0.05	0.09
Aeromonadales	AZ-CTR-LC	19.96±10.46%	5.70±10.39%	15.70±21.11%	0.7	0.37	0.06
Lactobacillales	AZ-CTR-LC	1.43±2.33%	0.99±0.99%	5.67±5.88%	0.17	0.12	0.71
Erysipelotrichales	AZ-CTR-LC	1.36±0.76%	4.96±4.51%	4.29±2.78%	0.05	0.79	0.12
Spirochaetales	AZ-CTR-LC	0.22±0.22%	0.44±0.67%	0.75±1.57%	0.48	0.7	0.5
Burkholderiales	AZ-CTR-LC	0.17±0.13%	0.32±0.20%	0.14±0.08%	0.7	0.11	0.2
Desulfovibrionales	AZ-CTR-LC	0.17±0.19%	0.00±0.00%	0.55±0.28%	0.04	0	0.09
Oceanospirillales	AZ-CTR-LC	0.17±0.36%	0.01±0.00%	0.07±0.06%	0.54	0.04	0.34
RF39	AZ-CTR-LC	0.16±0.11%	0.49±0.43%	0.37±0.50%	0.38	0.69	0.13
YS2	AZ-CTR-LC	0.15±0.12%	0.03±0.06%	0.18±0.17%	0.74	0.1	0.08
Chlamydiales	AZ-CTR-LC	0.08±0.06%	0.30±0.52%	0.30±0.54%	0.38	0.99	0.37
Turicibacterales	AZ-CTR-LC	0.06±0.04%	0.07±0.09%	0.12±0.15%	0.4	0.49	0.91
Campylobacterales	AZ-CTR-LC	0.03±0.01%	0.02±0.04%	0.32±0.37%	0.12	0.11	0.77
Enterobacteriales	AZ-CTR-LC	0.02±0.02%	0.07±0.08%	3.05±6.46%	0.33	0.33	0.21
Coriobacteriales	AZ-CTR-LC	0.01±0.01%	0.02±0.01%	0.06±0.06%	0.08	0.13	0.13
Cytophagales	AZ-CTR-LC	0.01±0.00%	0.10±0.11%	0.02±0.02%	0.09	0.17	0.09
Sphingobacteriales	AZ-CTR-LC	0.01±0.01%	0.02±0.02%	0.01±0.01%	0.41	0.29	0.09
Rhizobiales	AZ-CTR-LC	0.01±0.01%	0.06±0.06%	0.03±0.02%	0.04	0.36	0.09
Tremblayales	AZ-CTR-LC	0.01±0.03%	6.13±7.46%	2.53±3.45%	0.14	0.36	0.1
Pasteurellales	AZ-CTR-LC	0.01±0.01%	0.32±0.47%	4.11±5.39%	0.13	0.16	0.18
Pseudomonadales	AZ-CTR-LC	0.01±0.00%	0.10±0.08%	0.05±0.02%	0.02	0.24	0.06
Xanthomonadales	AZ-CTR-LC	0.01±0.01%	0.08±0.07%	0.04±0.04%	0.11	0.32	0.07
CW040	AZ-CTR-LC	0.01±0.01%	0.00±0.00%	0.01±0.01%	0.77	0.26	0.24
WCHB1-41	AZ-CTR-LC	0.01±0.02%	0.00±0.00%	0.06±0.08%	0.22	0.13	0.26
Verrucomicrobiales	AZ-CTR-LC	0.01±0.00%	0.05±0.04%	0.03±0.02%	0.04	0.36	0.06
iii1-15	AZ-CTR-LC	0.00±0.00%	0.01±0.01%	0.00±0.00%	0.32	0.2	0.12
Acidobacteriales	AZ-CTR-LC	0.00±0.00%	0.00±0.00%	0.00±0.00%	0.83	0.23	0.27
RB41	AZ-CTR-LC	0.00±0.00%	0.02±0.01%	0.01±0.01%	0.02	0.31	0.05
Actinomycetales	AZ-CTR-LC	0.00±0.00%	0.06±0.10%	0.02±0.01%	0.04	0.32	0.22
Flavobacteriales	AZ-CTR-LC	0.00±0.00%	0.02±0.02%	0.01±0.01%	0.17	0.28	0.07
[Saprospirales]	AZ-CTR-LC	0.00±0.00%	0.04±0.03%	0.02±0.01%	0.03	0.16	0.02
Streptophyta	AZ-CTR-LC	0.00±0.00%	0.00±0.01%	0.00±0.00%	0.85	0.41	0.39
Deferribacterales	AZ-CTR-LC	0.00±0.00%	0.00±0.00%	0.00±0.01%	0.44	0.53	0.7
Bacillales	AZ-CTR-LC	0.00±0.00%	0.01±0.01%	0.01±0.00%	0.1	0.17	0.06
Nitrospirales	AZ-CTR-LC	0.00±0.00%	0.00±0.00%	0.01±0.01%	0.13	0.44	0.03
Caulobacterales	AZ-CTR-LC	0.00±0.00%	0.01±0.01%	0.01±0.00%	0.01	0.15	0.02
Sphingomonadales	AZ-CTR-LC	0.00±0.00%	0.02±0.01%	0.01±0.01%	0.05	0.09	0.01
Neisseriales	AZ-CTR-LC	0.00±0.00%	0.00±0.00%	0.00±0.00%	0.55	0.68	0.97
Rhodocyclales	AZ-CTR-LC	0.00±0.00%	0.02±0.02%	0.05±0.05%	0.05	0.24	0.03
SC-I-84	AZ-CTR-LC	0.00±0.00%	0.01±0.01%	0.00±0.00%	0.31	0.14	0.05
Sphaerochaetales	AZ-CTR-LC	0.00±0.00%	0.01±0.01%	0.00±0.00%	0.99	0.2	0.22
[Chthoniobacterales]	AZ-CTR-LC	0.00±0.00%	0.03±0.02%	0.01±0.01%	0.17	0.15	0.03
DS-100	AZ-CTR	0.00±0.00%	0.00±0.00%	0.00±0.00%	0.35	0.37	0.23
DS-18	AZ-CTR	0.00±0.00%	0.00±0.00%	0.00±0.00%	0.35	0.19	0.08
Bifidobacteriales	AZ-CTR	0.00±0.00%	0.00±0.00%	0.03±0.06%	0.34	0.39	0.1
Fusobacteriales	AZ-CTR	0.00±0.00%	0.00±0.00%	0.10±0.20%	0.29	0.29	0.35
Ellin5290	AZ-CTR	0.00±0.00%	0.00±0.00%	0.00±0.00%	0.35	0.56	0.15
Gemmatimonadales	AZ-CTR	0.00±0.00%	0.01±0.01%	0.00±0.00%	0.1	0.12	0.03
N1423WL	AZ-CTR	0.00±0.00%	0.00±0.00%	0.00±0.00%	0.05	0.82	0.22
Rhodospirillales	AZ-CTR	0.00±0.00%	0.01±0.03%	0.00±0.00%	0.04	0.47	0.29
Ellin6067	AZ-CTR	0.00±0.00%	0.00±0.00%	0.00±0.00%	0.35	0.16	0.09
Desulfuromonadales	AZ-CTR	0.00±0.00%	0.01±0.02%	0.00±0.00%	0.35	0.32	0.29
Syntrophobacterales	AZ-CTR	0.00±0.00%	0.00±0.00%	0.00±0.00%	0.35	0.76	0.18
GMD14H09	CTR-LC	0.02±0.04%	0.00±0.00%	0.34±0.70%	0.34	0.31	0.31
Synergistales	CTR-LC	0.01±0.01%	0.00±0.00%	0.02±0.02%	0.29	0.09	0.14
Fibrobacterales	CTR-LC	0.01±0.01%	0.00±0.00%	0.00±0.01%	0.5	0.28	0.09
MND1	AZ	0.00±0.00%	0.00±0.01%	0.00±0.00%	1	0.21	0.21
Chloroflexales	AZ	0.00±0.00%	0.00±0.01%	0.00±0.00%	1	0.28	0.28
Herpetosiphonales	AZ	0.00±0.00%	0.00±0.01%	0.00±0.00%	1	0.35	0.35
Rhodobacterales	AZ	0.00±0.00%	0.00±0.01%	0.00±0.00%	1	0.35	0.35
Desulfobacterales	AZ	0.00±0.00%	0.01±0.01%	0.00±0.00%	1	0.23	0.23
Alteromonadales	AZ	0.00±0.00%	0.00±0.01%	0.00±0.00%	1	0.26	0.26
Sediment-1	AZ	0.00±0.00%	0.01±0.01%	0.00±0.00%	1	0.27	0.27
Opitutales	AZ	0.00±0.00%	0.00±0.00%	0.00±0.00%	1	0.08	0.08
Deinococcales	AZ	0.00±0.00%	0.00±0.00%	0.00±0.00%	1	0.18	0.18
Pirellulales	CTR	0.00±0.00%	0.00±0.00%	0.01±0.01%	0.1	0.1	1
Anaeroplasmatales	CTR	0.00±0.00%	0.00±0.00%	0.02±0.04%	0.35	0.35	1
Unassigned	AZ-CTR-LC	0.64±0.25%	2.67±4.16%	0.87±0.69%	0.52	0.37	0.31

Table S9 | The family composition among AZ, LC and CTR (n=5 per group).

Family	Group	LC	AZ	CTR	P (LC vs CTR)	P (AZ vs CTR)	P (LC vs AZ)
Prevotellaceae	AZ-CTR-LC	30.17±7.58%	24.65±12.59%	10.41±5.14%	0	0.05	0.43
Succinivibrionaceae	AZ-CTR-LC	23.55±7.74%	1.10±1.67%	6.41±4.29%	0.01	0.06	0
Veillonellaceae	AZ-CTR-LC	22.93±7.23%	4.25±2.73%	19.20±6.30%	0.44	0	0
[Paraprevotellaceae]	AZ-CTR-LC	5.67±1.89%	11.93±4.74%	7.29±7.13%	0.64	0.26	0.03
Ruminococcaceae	AZ-CTR-LC	5.56±3.06%	7.37±2.56%	11.10±6.47%	0.12	0.26	0.34
Lachnospiraceae	AZ-CTR-LC	2.62±1.21%	3.84±2.52%	3.45±3.44%	0.62	0.84	0.36
S24-7	AZ-CTR-LC	1.77±0.82%	10.20±5.57%	3.23±1.59%	0.1	0.03	0.01
Lactobacillaceae	AZ-CTR-LC	1.39±2.35%	0.96±0.98%	5.61±5.84%	0.17	0.12	0.72
Erysipelotrichaceae	AZ-CTR-LC	1.36±0.76%	4.96±4.51%	4.29±2.78%	0.05	0.79	0.12
Porphyromonadaceae	AZ-CTR-LC	1.16±1.81%	3.06±2.89%	0.19±0.17%	0.27	0.06	0.25
Clostridiaceae	AZ-CTR-LC	0.59±0.28%	1.18±0.80%	1.48±1.69%	0.34	0.73	0.2
Spirochaetaceae	AZ-CTR-LC	0.22±0.22%	0.44±0.67%	0.75±1.57%	0.48	0.7	0.5
Desulfovibrionaceae	AZ-CTR-LC	0.17±0.19%	0.00±0.00%	0.55±0.28%	0.04	0	0.09
Halomonadaceae	AZ-CTR-LC	0.17±0.36%	0.01±0.00%	0.07±0.06%	0.54	0.04	0.34
Alcaligenaceae	AZ-CTR-LC	0.13±0.14%	0.06±0.09%	0.03±0.02%	0.18	0.49	0.42
Peptococcaceae	AZ-CTR-LC	0.09±0.14%	0.03±0.05%	0.15±0.20%	0.6	0.2	0.33
Chlamydiaceae	AZ-CTR-LC	0.08±0.06%	0.30±0.52%	0.30±0.54%	0.38	0.99	0.37
RF16	AZ-CTR-LC	0.06±0.09%	0.04±0.07%	0.24±0.53%	0.47	0.43	0.74
Turicibacteraceae	AZ-CTR-LC	0.06±0.04%	0.07±0.09%	0.12±0.15%	0.4	0.49	0.91
Peptostreptococcaceae	AZ-CTR-LC	0.05±0.03%	0.18±0.22%	0.18±0.26%	0.29	0.98	0.2
Streptococcaceae	AZ-CTR-LC	0.04±0.04%	0.03±0.03%	0.06±0.06%	0.56	0.24	0.46
Bacteroidaceae	AZ-CTR-LC	0.03±0.03%	0.10±0.14%	0.23±0.49%	0.39	0.58	0.31
Oxalobacteraceae	AZ-CTR-LC	0.03±0.03%	0.16±0.12%	0.07±0.04%	0.1	0.2	0.06
Helicobacteraceae	AZ-CTR-LC	0.02±0.02%	0.02±0.04%	0.01±0.01%	0.2	0.33	0.78
Enterobacteriaceae	AZ-CTR-LC	0.02±0.02%	0.07±0.08%	3.05±6.46%	0.33	0.33	0.21
Coriobacteriaceae	AZ-CTR-LC	0.01±0.01%	0.02±0.01%	0.06±0.06%	0.08	0.13	0.13
Cytophagaceae	AZ-CTR-LC	0.01±0.00%	0.10±0.11%	0.02±0.02%	0.09	0.17	0.09
Sphingobacteriaceae	AZ-CTR-LC	0.01±0.01%	0.02±0.02%	0.01±0.01%	0.41	0.29	0.09
[Mogibacteriaceae]	AZ-CTR-LC	0.01±0.01%	0.03±0.02%	0.10±0.10%	0.07	0.15	0.1
Christensenellaceae	AZ-CTR-LC	0.01±0.01%	0.02±0.01%	1.00±2.12%	0.33	0.33	0.15
Burkholderiaceae	AZ-CTR-LC	0.01±0.00%	0.04±0.03%	0.02±0.02%	0.1	0.28	0.04
Pasteurellaceae	AZ-CTR-LC	0.01±0.01%	0.32±0.47%	4.11±5.39%	0.13	0.16	0.18
Pseudomonadaceae	AZ-CTR-LC	0.01±0.01%	0.09±0.08%	0.03±0.02%	0.1	0.14	0.06
Xanthomonadaceae	AZ-CTR-LC	0.01±0.01%	0.08±0.07%	0.04±0.04%	0.11	0.32	0.07
F16	AZ-CTR-LC	0.01±0.01%	0.00±0.00%	0.01±0.01%	0.77	0.26	0.24
RFP12	AZ-CTR-LC	0.01±0.02%	0.00±0.00%	0.06±0.08%	0.22	0.13	0.26
Verrucomicrobiaceae	AZ-CTR-LC	0.01±0.00%	0.05±0.04%	0.03±0.02%	0.04	0.36	0.06
Ellin6075	AZ-CTR-LC	0.00±0.00%	0.02±0.01%	0.01±0.00%	0.05	0.25	0.06
Acidobacteriaceae	AZ-CTR-LC	0.00±0.00%	0.00±0.00%	0.00±0.00%	0.83	0.23	0.27
Actinomycetaceae	AZ-CTR-LC	0.00±0.00%	0.00±0.00%	0.00±0.00%	0.19	0.61	0.06
Micrococcaceae	AZ-CTR-LC	0.00±0.00%	0.00±0.00%	0.01±0.01%	0.41	0.37	0.98
Chitinophagaceae	AZ-CTR-LC	0.00±0.00%	0.04±0.03%	0.02±0.01%	0.03	0.16	0.02
Rikenellaceae	AZ-CTR-LC	0.00±0.00%	0.00±0.00%	0.05±0.11%	0.35	0.36	0.42
[Weeksellaceae]	AZ-CTR-LC	0.00±0.00%	0.01±0.02%	0.01±0.01%	0.2	0.4	0.15
Flavobacteriaceae	AZ-CTR-LC	0.00±0.00%	0.01±0.01%	0.00±0.00%	0.16	0.13	0.01
Deferribacteraceae	AZ-CTR-LC	0.00±0.00%	0.00±0.00%	0.00±0.01%	0.44	0.53	0.7
Bacillaceae	AZ-CTR-LC	0.00±0.00%	0.01±0.01%	0.01±0.00%	0.08	0.26	0.06

Staphylococcaceae	AZ-CTR-LC	0.00±0.00%	0.00±0.00%	0.00±0.00%	0.65	0.07	0.12
Gracilibacteraceae	AZ-CTR-LC	0.00±0.00%	0.00±0.00%	0.00±0.00%	0.44	0.27	0.49
0319-6A21	AZ-CTR-LC	0.00±0.00%	0.00±0.00%	0.01±0.01%	0.13	0.44	0.03
Caulobacteraceae	AZ-CTR-LC	0.00±0.00%	0.01±0.01%	0.01±0.00%	0.01	0.15	0.02
Bradyrhizobiaceae	AZ-CTR-LC	0.00±0.00%	0.01±0.01%	0.01±0.00%	0.02	0.12	0.03
Brucellaceae	AZ-CTR-LC	0.00±0.00%	0.00±0.00%	0.00±0.00%	0.18	0.69	0.33
Hyphomicrobiaceae	AZ-CTR-LC	0.00±0.00%	0.01±0.00%	0.01±0.00%	0.47	1	0.5
Phyllobacteriaceae	AZ-CTR-LC	0.00±0.00%	0.00±0.00%	0.00±0.00%	0.67	0.53	0.19
Rhizobiaceae	AZ-CTR-LC	0.00±0.00%	0.02±0.02%	0.01±0.01%	0.08	0.42	0.07
Sphingomonadaceae	AZ-CTR-LC	0.00±0.00%	0.02±0.01%	0.01±0.01%	0.05	0.09	0.01
Comamonadaceae	AZ-CTR-LC	0.00±0.00%	0.07±0.08%	0.01±0.01%	0.09	0.18	0.11
Neisseriaceae	AZ-CTR-LC	0.00±0.00%	0.00±0.00%	0.00±0.00%	0.55	0.68	0.97
Rhodocyclaceae	AZ-CTR-LC	0.00±0.00%	0.02±0.02%	0.05±0.05%	0.05	0.24	0.03
Moraxellaceae	AZ-CTR-LC	0.00±0.00%	0.00±0.00%	0.02±0.02%	0.11	0.13	0.82
Sphaerochaetaceae	AZ-CTR-LC	0.00±0.00%	0.01±0.01%	0.00±0.00%	0.99	0.2	0.22
[Chthoniobacteraceae]	AZ-CTR-LC	0.00±0.00%	0.03±0.02%	0.01±0.01%	0.17	0.15	0.03
RB40	AZ-CTR	0.00±0.00%	0.00±0.00%	0.00±0.00%	0.14	0.39	0.14
Nocardiaceae	AZ-CTR	0.00±0.00%	0.00±0.00%	0.00±0.00%	0.02	0.04	0.35
Nocardioidaceae	AZ-CTR	0.00±0.00%	0.04±0.06%	0.00±0.00%	0.02	0.23	0.19
Bifidobacteriaceae	AZ-CTR	0.00±0.00%	0.00±0.00%	0.03±0.06%	0.34	0.39	0.1
p-2534-18B5	AZ-CTR	0.00±0.00%	0.00±0.00%	0.16±0.36%	0.34	0.35	0.13
Dehalobacteriaceae	AZ-CTR	0.00±0.00%	0.00±0.00%	0.01±0.02%	0.23	0.25	0.35
Fusobacteriaceae	AZ-CTR	0.00±0.00%	0.00±0.00%	0.10±0.20%	0.28	0.29	0.35
Ellin5301	AZ-CTR	0.00±0.00%	0.01±0.01%	0.00±0.00%	0.1	0.12	0.03
Desulfuromonadaceae	AZ-CTR	0.00±0.00%	0.01±0.02%	0.00±0.00%	0.35	0.32	0.29
Syntrophobacteraceae	AZ-CTR	0.00±0.00%	0.00±0.00%	0.00±0.00%	0.35	0.76	0.18
Corynebacteriaceae	AZ-LC	0.00±0.00%	0.00±0.01%	0.00±0.00%	0.35	0.18	0.22
Fibrobacteraceae	CTR-LC	0.01±0.01%	0.00±0.00%	0.00±0.01%	0.5	0.28	0.09
Campylobacteraceae	CTR-LC	0.01±0.02%	0.00±0.00%	0.31±0.37%	0.11	0.1	0.16
Dethiosulfovibrionaceae	CTR-LC	0.01±0.01%	0.00±0.00%	0.01±0.01%	0.41	0.07	0.14
Deinococcaceae	AZ	0.00±0.00%	0.00±0.00%	0.00±0.00%	1	0.18	0.18
Kineosporiaceae	AZ	0.00±0.00%	0.01±0.02%	0.00±0.00%	1	0.35	0.35
Micromonosporaceae	AZ	0.00±0.00%	0.00±0.01%	0.00±0.00%	1	0.35	0.35
Chloroflexaceae	AZ	0.00±0.00%	0.00±0.01%	0.00±0.00%	1	0.28	0.28
JTB215	AZ	0.00±0.00%	0.00±0.01%	0.00±0.00%	1	0.27	0.27
Methylobacteriaceae	AZ	0.00±0.00%	0.01±0.02%	0.00±0.00%	1	0.32	0.32
Rhodobacteraceae	AZ	0.00±0.00%	0.00±0.01%	0.00±0.00%	1	0.35	0.35
Rhodospirillaceae	AZ	0.00±0.00%	0.01±0.03%	0.00±0.00%	1	0.29	0.29
Desulfobacteraceae	AZ	0.00±0.00%	0.01±0.01%	0.00±0.00%	1	0.23	0.23
Alteromonadaceae	AZ	0.00±0.00%	0.00±0.01%	0.00±0.00%	1	0.26	0.26
Opitutaceae	AZ	0.00±0.00%	0.00±0.00%	0.00±0.00%	1	0.08	0.08
PRR-10	AZ	0.00±0.00%	0.01±0.01%	0.00±0.00%	1	0.27	0.27
[Barnesiellaceae]	CTR	0.00±0.00%	0.00±0.00%	0.00±0.01%	0.26	0.26	1
[Odoribacteraceae]	CTR	0.00±0.00%	0.00±0.00%	0.00±0.00%	0.14	0.14	1
Leptotrichiaceae	CTR	0.00±0.00%	0.00±0.00%	0.00±0.01%	0.35	0.35	1
Pirellulaceae	CTR	0.00±0.00%	0.00±0.00%	0.01±0.01%	0.1	0.1	1
Acetobacteraceae	CTR	0.00±0.00%	0.00±0.00%	0.00±0.00%	0.04	0.04	1
Synergistaceae	CTR	0.00±0.00%	0.00±0.00%	0.00±0.01%	0.35	0.35	1
Anaeroplasmataceae	CTR	0.00±0.00%	0.00±0.00%	0.02±0.04%	0.35	0.35	1
Unassigned	AZ-CTR-LC	0.64±0.25%	2.67±4.16%	0.87±0.69%	0.52	0.37	0.31

Table S10 | The genus composition among AZ, LC and CTR (n=5 per group).

Genus	Group	LC	AZ	CTR	P (LC vs CTR)	P (AZ vs CTR)	P (LC vs AZ)
Prevotella	AZ-CTR-LC	30.17±7.58%	29.13±8.80%	10.41±5.14%	0	0.01	0.85
Succinivibrio	AZ-CTR-LC	23.27±7.76%	1.08±1.68%	6.23±4.21%	0.01	0.06	0
Anaerovibrio	AZ-CTR-LC	8.87±5.56%	1.89±2.17%	5.41±3.20%	0.26	0.08	0.03
[Prevotella]	AZ-CTR-LC	4.44±2.11%	9.04±5.20%	6.25±7.16%	0.6	0.5	0.1
Oscillospira	AZ-CTR-LC	2.23±0.97%	2.02±0.72%	1.75±0.75%	0.41	0.58	0.7
Mitsuokella	AZ-CTR-LC	1.85±1.86%	0.36±0.51%	1.35±1.76%	0.68	0.26	0.12
Lactobacillus	AZ-CTR-LC	1.39±2.35%	0.96±0.98%	5.61±5.84%	0.17	0.12	0.72
Parabacteroides	AZ-CTR-LC	1.16±1.81%	3.06±2.89%	0.17±0.13%	0.26	0.06	0.25
Megasphaera	AZ-CTR-LC	1.15±1.89%	0.72±1.00%	5.04±4.03%	0.09	0.05	0.67
CF231	AZ-CTR-LC	1.03±0.36%	2.76±1.50%	0.76±0.71%	0.47	0.03	0.04
p-75-a5	AZ-CTR-LC	0.95±0.62%	4.33±4.68%	3.38±2.51%	0.07	0.7	0.15
Phascolarctobacterium	AZ-CTR-LC	0.73±0.40%	0.77±0.32%	1.52±0.85%	0.1	0.1	0.87
Faecalibacterium	AZ-CTR-LC	0.56±0.58%	0.79±0.62%	0.41±0.21%	0.59	0.23	0.57
Ruminococcus	AZ-CTR-LC	0.39±0.31%	0.39±0.17%	0.35±0.53%	0.88	0.86	0.99
Lachnospira	AZ-CTR-LC	0.34±0.22%	0.13±0.16%	0.32±0.40%	0.92	0.37	0.14
Acidaminococcus	AZ-CTR-LC	0.32±0.47%	0.20±0.28%	0.65±0.47%	0.3	0.11	0.64
Selenomonas	AZ-CTR-LC	0.31±0.30%	0.17±0.32%	0.46±0.84%	0.71	0.48	0.48
Clostridium	AZ-CTR-LC	0.24±0.17%	0.42±0.28%	0.61±0.72%	0.29	0.59	0.25
Treponema	AZ-CTR-LC	0.22±0.22%	0.44±0.67%	0.75±1.57%	0.48	0.7	0.5
Roseburia	AZ-CTR-LC	0.17±0.12%	0.09±0.05%	0.11±0.07%	0.38	0.62	0.22
Halomonas	AZ-CTR-LC	0.17±0.36%	0.01±0.00%	0.07±0.06%	0.54	0.04	0.34
Desulfovibrio	AZ-CTR-LC	0.16±0.18%	0.00±0.00%	0.54±0.27%	0.03	0	0.1
Sutterella	AZ-CTR-LC	0.13±0.14%	0.06±0.09%	0.03±0.01%	0.18	0.51	0.4
Blautia	AZ-CTR-LC	0.10±0.07%	0.27±0.20%	0.22±0.26%	0.36	0.72	0.11
Bulleidia	AZ-CTR-LC	0.10±0.09%	0.19±0.13%	0.25±0.11%	0.04	0.5	0.22
rc4-4	AZ-CTR-LC	0.09±0.13%	0.02±0.03%	0.13±0.20%	0.72	0.27	0.29
Chlamydia	AZ-CTR-LC	0.08±0.06%	0.30±0.52%	0.30±0.52%	0.38	1	0.37
Turicibacter	AZ-CTR-LC	0.06±0.04%	0.07±0.09%	0.12±0.15%	0.4	0.49	0.91
[Eubacterium]	AZ-CTR-LC	0.06±0.05%	0.14±0.16%	0.14±0.11%	0.17	0.95	0.35
RFN20	AZ-CTR-LC	0.06±0.03%	0.02±0.02%	0.03±0.03%	0.14	0.34	0.03
Streptococcus	AZ-CTR-LC	0.04±0.04%	0.03±0.03%	0.06±0.06%	0.58	0.22	0.41
Coprococcus	AZ-CTR-LC	0.04±0.06%	0.07±0.05%	0.14±0.16%	0.26	0.39	0.49
Bacteroides	AZ-CTR-LC	0.03±0.03%	0.10±0.14%	0.23±0.49%	0.39	0.58	0.31
L7A_E11	AZ-CTR-LC	0.03±0.01%	0.10±0.10%	0.12±0.11%	0.1	0.76	0.16
YRC22	AZ-CTR-LC	0.02±0.05%	0.02±0.05%	0.06±0.11%	0.57	0.58	0.98
Veillonella	AZ-CTR-LC	0.02±0.02%	0.02±0.05%	0.43±0.93%	0.35	0.36	0.77
Helicobacter	AZ-CTR-LC	0.02±0.02%	0.02±0.04%	0.01±0.01%	0.2	0.33	0.78
Anaerostipes	AZ-CTR-LC	0.01±0.01%	0.01±0.01%	0.02±0.04%	0.52	0.51	0.96
Dorea	AZ-CTR-LC	0.01±0.01%	0.06±0.04%	0.05±0.02%	0.02	0.36	0.01
Catenibacterium	AZ-CTR-LC	0.01±0.00%	0.00±0.00%	0.03±0.02%	0.05	0.04	0.35
Oxalobacter	AZ-CTR-LC	0.01±0.02%	0.00±0.00%	0.01±0.01%	0.65	0.25	0.31
Ralstonia	AZ-CTR-LC	0.01±0.01%	0.14±0.11%	0.06±0.05%	0.06	0.18	0.03
Actinobacillus	AZ-CTR-LC	0.01±0.00%	0.31±0.47%	3.98±5.24%	0.13	0.16	0.18
Pseudomonas	AZ-CTR-LC	0.01±0.01%	0.09±0.08%	0.03±0.02%	0.1	0.14	0.06
Terriglobus	AZ-CTR-LC	0.00±0.00%	0.00±0.00%	0.00±0.00%	0.83	0.23	0.27
Actinomyces	AZ-CTR-LC	0.00±0.00%	0.00±0.00%	0.00±0.00%	0.19	0.61	0.06
Collinsella	AZ-CTR-LC	0.00±0.00%	0.00±0.00%	0.01±0.01%	0.02	0.04	0.32
Slackia	AZ-CTR-LC	0.00±0.00%	0.00±0.00%	0.00±0.00%	0.59	0.28	0.38
Chitinophaga	AZ-CTR-LC	0.00±0.00%	0.01±0.01%	0.01±0.01%	0.05	0.19	0.01
Flavisolibacter	AZ-CTR-LC	0.00±0.00%	0.02±0.02%	0.01±0.01%	0.39	0.14	0.08
Dyadobacter	AZ-CTR-LC	0.00±0.00%	0.05±0.05%	0.02±0.02%	0.06	0.24	0.05
Chryseobacterium	AZ-CTR-LC	0.00±0.00%	0.01±0.02%	0.01±0.01%	0.2	0.4	0.15
Flavobacterium	AZ-CTR-LC	0.00±0.00%	0.01±0.01%	0.00±0.00%	0.16	0.13	0.01
Pedobacter	AZ-CTR-LC	0.00±0.00%	0.00±0.00%	0.00±0.00%	0.12	0.88	0.17
Mucispirillum	AZ-CTR-LC	0.00±0.00%	0.00±0.00%	0.00±0.01%	0.44	0.53	0.7
Bacillus	AZ-CTR-LC	0.00±0.00%	0.01±0.01%	0.01±0.00%	0.08	0.26	0.06
Staphylococcus	AZ-CTR-LC	0.00±0.00%	0.00±0.00%	0.01±0.00%	0.65	0.07	0.12
[Ruminococcus]	AZ-CTR-LC	0.00±0.00%	0.01±0.01%	0.02±0.02%	0.3	0.92	0.08
Peptococcus	AZ-CTR-LC	0.00±0.00%	0.01±0.01%	0.03±0.02%	0.04	0.12	0.49
Dialister	AZ-CTR-LC	0.00±0.00%	0.02±0.04%	0.01±0.02%	0.47	0.62	0.4
Sharpea	AZ-CTR-LC	0.00±0.00%	0.01±0.01%	0.02±0.05%	0.38	0.55	0.15

Balneimonas	AZ-CTR-LC	0.00±0.00%	0.00±0.00%	0.00±0.00%	0.5	0.39	0.26
Bradyrhizobium	AZ-CTR-LC	0.00±0.00%	0.01±0.01%	0.00±0.00%	0	0.17	0.01
Ochrobactrum	AZ-CTR-LC	0.00±0.00%	0.00±0.00%	0.00±0.00%	0.18	0.69	0.33
Devosia	AZ-CTR-LC	0.00±0.00%	0.00±0.00%	0.00±0.00%	0.46	0.43	0.89
Rhodoplanes	AZ-CTR-LC	0.00±0.00%	0.00±0.00%	0.00±0.00%	0.95	0.16	0.14
Mesorhizobium	AZ-CTR-LC	0.00±0.00%	0.00±0.00%	0.00±0.00%	0.67	0.53	0.19
Agrobacterium	AZ-CTR-LC	0.00±0.00%	0.00±0.00%	0.00±0.00%	0.95	0.23	0.22
Rhizobium	AZ-CTR-LC	0.00±0.00%	0.02±0.02%	0.01±0.01%	0.05	0.61	0.13
Kaistobacter	AZ-CTR-LC	0.00±0.00%	0.00±0.00%	0.00±0.00%	0.4	0.32	0.05
Sphingobium	AZ-CTR-LC	0.00±0.00%	0.01±0.01%	0.01±0.00%	0.07	0.08	0
Sphingomonas	AZ-CTR-LC	0.00±0.00%	0.01±0.01%	0.00±0.00%	0.13	0.22	0.09
Burkholderia	AZ-CTR-LC	0.00±0.00%	0.03±0.02%	0.02±0.02%	0.1	0.3	0.02
Pandoraea	AZ-CTR-LC	0.00±0.00%	0.00±0.00%	0.00±0.00%	0.95	0.33	0.3
Variovorax	AZ-CTR-LC	0.00±0.00%	0.01±0.01%	0.00±0.00%	0.14	0.39	0.12
Cupriavidus	AZ-CTR-LC	0.00±0.00%	0.01±0.00%	0.00±0.00%	0.8	0.03	0.02
Pasteurella	AZ-CTR-LC	0.00±0.00%	0.01±0.01%	0.10±0.14%	0.17	0.19	0.09
Acinetobacter	AZ-CTR-LC	0.00±0.00%	0.00±0.00%	0.02±0.02%	0.11	0.13	0.82
Dokdonella	AZ-CTR-LC	0.00±0.00%	0.00±0.00%	0.00±0.00%	0.18	0.1	0.36
Luteimonas	AZ-CTR-LC	0.00±0.00%	0.00±0.00%	0.00±0.00%	0.31	0.98	0.27
Stenotrophomonas	AZ-CTR-LC	0.00±0.00%	0.01±0.01%	0.01±0.01%	0.34	0.57	0.15
Sphaerochaeta	AZ-CTR-LC	0.00±0.00%	0.01±0.01%	0.00±0.00%	0.99	0.2	0.22
Candidatus Xiphinematobacter	AZ-CTR-LC	0.00±0.00%	0.02±0.01%	0.01±0.01%	0.33	0.15	0.03
DA101	AZ-CTR-LC	0.00±0.00%	0.00±0.01%	0.00±0.00%	0.04	0.92	0.18
Akkermansia	AZ-CTR-LC	0.00±0.00%	0.00±0.00%	0.00±0.01%	0.53	0.66	0.78
Luteolibacter	AZ-CTR-LC	0.00±0.00%	0.04±0.03%	0.02±0.02%	0.07	0.42	0.05
Rhodococcus	AZ-CTR	0.00±0.00%	0.00±0.00%	0.00±0.00%	0.02	0.04	0.35
Aeromicrobium	AZ-CTR	0.00±0.00%	0.01±0.01%	0.00±0.00%	0.14	0.21	0.14
Nocardioides	AZ-CTR	0.00±0.00%	0.00±0.01%	0.00±0.00%	0.15	0.24	0.15
Bifidobacterium	AZ-CTR	0.00±0.00%	0.00±0.00%	0.03±0.06%	0.34	0.39	0.1
Niabella	AZ-CTR	0.00±0.00%	0.00±0.00%	0.00±0.00%	0.09	0.38	0.05
Sediminibacterium	AZ-CTR	0.00±0.00%	0.01±0.00%	0.00±0.00%	0.14	0.25	0.02
Lactococcus	AZ-CTR	0.00±0.00%	0.00±0.00%	0.00±0.00%	0.15	0.4	0.09
SMB53	AZ-CTR	0.00±0.00%	0.00±0.00%	0.00±0.00%	0.05	1	0.21
Dehalobacterium	AZ-CTR	0.00±0.00%	0.00±0.00%	0.01±0.02%	0.23	0.25	0.35
Tepidibacter	AZ-CTR	0.00±0.00%	0.00±0.00%	0.00±0.00%	0.17	0.06	0
Coprobacillus	AZ-CTR	0.00±0.00%	0.00±0.00%	0.00±0.01%	0.25	0.4	0.35
Fusobacterium	AZ-CTR	0.00±0.00%	0.00±0.00%	0.10±0.20%	0.28	0.29	0.35
Phenylobacterium	AZ-CTR	0.00±0.00%	0.01±0.01%	0.00±0.00%	0.01	0.74	0.1
Azohydromonas	AZ-CTR	0.00±0.00%	0.00±0.00%	0.00±0.00%	0.16	0.43	0.04
Desulfuromonas	AZ-CTR	0.00±0.00%	0.01±0.02%	0.00±0.00%	0.35	0.32	0.29
Pseudoxanthomonas	AZ-CTR	0.00±0.00%	0.00±0.00%	0.00±0.00%	0.35	0.55	0.12
Corynebacterium	AZ-LC	0.00±0.00%	0.00±0.01%	0.00±0.00%	0.35	0.18	0.22
Fibrobacter	CTR-LC	0.01±0.01%	0.00±0.00%	0.00±0.01%	0.5	0.28	0.09
Campylobacter	CTR-LC	0.01±0.02%	0.00±0.00%	0.31±0.37%	0.11	0.1	0.16
Deinococcus	AZ	0.00±0.00%	0.00±0.00%	0.00±0.00%	1	0.18	0.18
Kineosporia	AZ	0.00±0.00%	0.00±0.01%	0.00±0.00%	1	0.35	0.35
Catenuloplanes	AZ	0.00±0.00%	0.00±0.01%	0.00±0.00%	1	0.35	0.35
Hymenobacter	AZ	0.00±0.00%	0.03±0.07%	0.00±0.00%	1	0.32	0.32
Chloronema	AZ	0.00±0.00%	0.00±0.01%	0.00±0.00%	1	0.28	0.28
Methylobacterium	AZ	0.00±0.00%	0.01±0.01%	0.00±0.00%	1	0.3	0.3
Skermanella	AZ	0.00±0.00%	0.01±0.03%	0.00±0.00%	1	0.29	0.29
Polaromonas	AZ	0.00±0.00%	0.01±0.02%	0.00±0.00%	1	0.35	0.35
Janthinobacterium	AZ	0.00±0.00%	0.00±0.00%	0.00±0.00%	1	0.16	0.16
Desulfococcus	AZ	0.00±0.00%	0.00±0.00%	0.00±0.00%	1	0.25	0.25
Marinobacter	AZ	0.00±0.00%	0.00±0.01%	0.00±0.00%	1	0.26	0.26
Opitutus	AZ	0.00±0.00%	0.00±0.00%	0.00±0.00%	1	0.08	0.08
Butyricimonas	CTR	0.00±0.00%	0.00±0.00%	0.00±0.00%	0.14	0.14	1
Paludibacter	CTR	0.00±0.00%	0.00±0.00%	0.02±0.04%	0.18	0.18	1
Leptotrichia	CTR	0.00±0.00%	0.00±0.00%	0.00±0.01%	0.35	0.35	1
Azoarcus	CTR	0.00±0.00%	0.00±0.00%	0.04±0.05%	0.11	0.11	1
Aggregatibacter	CTR	0.00±0.00%	0.00±0.00%	0.01±0.02%	0.16	0.16	1
Anaeroplasma	CTR	0.00±0.00%	0.00±0.00%	0.02±0.04%	0.35	0.35	1
Sphingobacterium	COS	0.00±0.00%	0.00±0.00%	0.00±0.00%	0.12	1	0.12
Unassigned	AZ-CTR-LC	0.64±0.25%	2.67±4.16%	0.87±0.69%	0.52	0.37	0.31

Figure S1 | Different KO terms involved in the metabolism of cofactors and vitamins. Out of 85 different KO terms, 62 (73%) were greater than 0.01% in any group and were shown. The KO term of riboflavin kinase (K11753), whose relative abundance were greater than 0.1% in all groups, enriched on the KEGG level 3 of Riboflavin metabolism.

Figure S2 | Different KO terms involved in the pathway of glycan biosynthesis and metabolism. Out of 34 different KO terms, 29 (85%) were greater than 0.01% in any group and were shown. The KO terms of penicillin-binding protein 1A (K05366), penicillin-binding protein (K07258), UDP-N-acetylglucosamine acyltransferase (K00677), alpha-L-fucosidase (K01206), hexosaminidase (K12373), whose relative abundance were greater than 0.1% in any group, enriched on the KEGG level 3 of Peptidoglycan biosynthesis, Lipopolysaccharide biosynthesis, Other glycan degradation, and Glycosaminoglycan degradation, respectively.
